# Hybrid multiscale forecasting of SRU sulfur gas concentrations using VMD CEEMDAN and optimized PatchTST

**DOI:** 10.1016/j.isci.2026.114986

**Published:** 2026-02-11

**Authors:** Wenzhe Sun, Longhao Li, Binglin Lu, Lijun Jiang, Jie Zhang

**Affiliations:** 1 School of Electrical and Electronic Engineering, Shandong University of Technology, Zibo, Shandong 255000, China; 2 Shandong Shanbo Electric Machinery Group Co., Ltd., Zibo, Shandong 255299, China

**Keywords:** Chemistry, Computer science, engineering

## Abstract

The sulfur recovery process in SRUs is highly nonlinear and non-stationary, making accurate forecasting of H_2_S and SO_2_ concentrations challenging yet crucial for efficient, low-carbon operation. Many existing models fail to handle multi-scale fluctuations, high-frequency noise, and complex variable couplings, limiting their accuracy. This study presents a multi-scale framework combining variational mode decomposition (VMD), complete ensemble empirical mode decomposition with adaptive noise (CEEMDAN), an enhanced patch time-series transformer with ProbSparse attention (PatchTST-PSA), and projection iterative modeling optimization (PIMO). VMD decomposes the concentration series into intrinsic mode functions, and CEEMDAN suppresses noise while preserving dynamics. PatchTST-PSA captures nonlinear variable interactions, while PIMO optimizes hyperparameters. Experiments on SRU data from an Italian refinery demonstrate that the framework provides improved results in RMSE, MAE, MAPE, and R^2^ compared to six baseline models, highlighting its robustness and industrial relevance.

## Highlights

• Dual-stage VMD-CEEMDAN separates trend and high-frequency disturbances
• Sample-entropy-guided reconstruction reduces noise and dimensional redundancy
• Probabilistic sparse attention in PatchTST retains key long-range dependencies
• PIMO jointly tunes hyperparameters, improving accuracy and stability

## Graphical abstract

**Figure:**
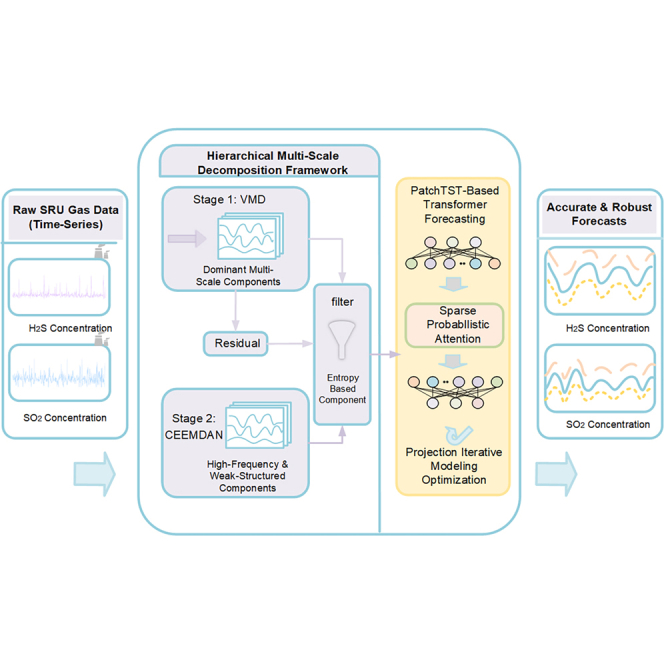

## Introduction

### Background

With the extensive development and utilization of sulfur-containing energy sources such as petroleum, natural gas, and coal, the emissions of sulfur compounds during industrial processes—including hydrogen sulfide (H_2_S) and sulfur dioxide (SO_2_)—continue to rise.^1^ These sulfur compounds contribute to significant atmospheric pollution and pose serious risks, including equipment corrosion and poisoning incidents, which threaten both production safety and human health. As a result, the efficient removal and resource recovery of these sulfur compounds have become critical tasks in industries such as petrochemicals,^2^ natural gas purification, and coal chemical processing.^3^

Among various desulfurization technologies, the sulfur recovery unit (SRU) plays a crucial role. SRUs typically employ the Claus process to convert hydrogen sulfide (H_2_S) into elemental sulfur, thereby achieving the dual objectives of pollution control and resource reutilization.^4^ During SRU operation, fluctuations in the concentrations of H_2_S and SO_2_ directly affect reaction heat balance, sulfur yield, and tail-gas emission quality. Therefore, accurate prediction of these key gas concentrations is essential for effective process control, improved sulfur recovery efficiency, and compliance with stringent environmental regulations.^5^

However, in practical SRU operations, the concentration signals of H_2_S and SO_2_ are characterized by strong non-stationarity arising from multiple temporal scales.^6^^,^^7^ Specifically, fast-scale fluctuations are mainly induced by measurement noise and valve switching, medium-scale variations are associated with operational adjustments, and slow-scale trends reflect load variations and catalyst aging. In this SRU context, multi-scale fluctuations refer to sulfur gas concentration dynamics composed of these heterogeneous timescale components, whose superposition poses significant challenges to conventional single-scale prediction models in terms of noise robustness and long-term dependency modeling.

### Literature review

Existing approaches for predicting sulfur gas concentrations in sulfur recovery units (SRUs) can be broadly classified into physical modeling and statistical modeling methods. Physical models are typically constructed based on mass conservation, thermodynamic equilibrium, and kinetic reaction mechanisms, offering strong interpretability and clear physical meaning.^8^ Representative studies include kinetic modeling of the Claus reactor and waste heat boiler,^9^ as well as thermodynamic-kinetic SRU models optimized through multi-objective strategies to improve sulfur recovery and steam production.^10^ Despite their interpretability, physical models generally require extensive prior knowledge, accurate parameter identification, and well-defined boundary conditions, which limits their flexibility and real-time applicability under varying industrial operating conditions. To alleviate these constraints, statistical modeling approaches have been introduced, leveraging historical process data to identify temporal patterns and perform regression-based predictions. Common techniques include autoregressive moving average (ARMA/ARIMA) models, vector autoregression (VAR), and multiple linear regression.^11^ However, these methods usually rely on assumptions of linearity and stationarity, making them inadequate for SRU gas concentration signals that exhibit strong nonlinearity and multi-scale temporal fluctuations.

However, sulfur gas concentration sequences in SRU processes typically exhibit strong nonlinearity, non-stationarity, and pronounced multi-scale temporal fluctuations, which makes conventional statistical models inadequate for accurately capturing their dynamic behaviors.^12^ To address these challenges, increasing attention has been devoted to artificial intelligence-based methods, particularly nonlinear modeling approaches based on machine learning and deep learning frameworks.^13^ Early studies employed shallow learning models, such as extreme learning machines (ELMs)^14^ and support vector machines (SVMs),^15^ to predict pollutant and sulfur gas concentrations. Subsequent research further improved predictive performance by incorporating feature learning mechanisms, for example, by integrating autoencoders with ELM.^16^ With the development of deep learning, hybrid architectures combining convolutional neural networks (CNNs) and recurrent neural networks (RNNs) have been widely adopted. Representative examples include PC CNN-GRU models for SO_2_ diffusion modeling,^17^ CNN-LSTM structures for capturing spatial-temporal dependencies in gas production processes,^18^^,^^19^ and related variants.

Despite their effectiveness, CNN- and RNN-based models often suffer from limited receptive fields or insufficient capability in modeling long-range temporal dependencies, especially under long sequences and complex noise conditions. The introduction of Transformer architectures^20^ significantly alleviated these limitations through self-attention mechanisms. More recently, PatchTST^21^ further enhanced Transformer-based time-series forecasting by mapping long sequences into patch-level tokens, improving sensitivity to local variations while retaining global modeling capacity, which makes it particularly suitable for SRU scenarios. Nevertheless, standard self-attention mechanisms in Transformers may still incur redundant computations and attention leakage under long-sequence and high-noise conditions. To overcome this limitation, this study incorporates sparse probabilistic self-attention into the PatchTST framework (PatchTST-PSA), which selectively focuses on dominant queries. This strategy preserves the original backbone structure while effectively capturing key long-range dependencies and local patterns, and simultaneously reduces computational complexity.

Recent years have witnessed rapid growth in hybrid forecasting frameworks that integrate signal decomposition, entropy analysis, and attention-based deep learning models for complex industrial and energy time series.^22^^,^^23^^,^^24^ In photovoltaic and wind power forecasting, decomposition techniques such as VMD and CEEMDAN are widely employed to mitigate non-stationarity and noise contamination, followed by attention-enhanced deep prediction models. Representative studies include VMD-SSA-Transformer-LSTM architectures for photovoltaic power forecasting, as well as entropy-guided secondary decomposition pipelines (e.g., EMD-VMD or CEEMDAN-VMD) combined with Autoformer or Informer models, which have demonstrated improved robustness under varying operating conditions and seasonal patterns.^23^^,^^24^^,^^25^

More recent studies have further extended this paradigm by introducing multi-stage decomposition and entropy-based component selection strategies, such as CEEMDAN-SE-HDBSCAN-VMD frameworks with parallel TCN-BiGRU predictors, as well as PSO-optimized VMD-SE-ICEEMDAN two-stage decomposition schemes coupled with attention-based Att-S2S models.^26^^,^^27^ While these hybrid approaches substantially improve feature separability and forecasting accuracy, most of them rely on RNN- or TCN-based predictors together with dense attention mechanisms, which may limit long-range dependency modeling and lead to increased computational overhead for long-horizon sequences. Moreover, secondary decomposition is typically applied to selected intrinsic mode functions, whereas residual-level disturbances remaining after primary decomposition are rarely addressed explicitly.^28^^,^^29^^,^^30^ In contrast, the proposed framework introduces a residual-oriented two-stage decomposition strategy (VDES) and integrates it with a PatchTST backbone equipped with sparse probabilistic attention, aiming to jointly enhance noise robustness, long-sequence modeling capability, and computational efficiency for sulfur-gas concentration forecasting in SRU processes.

Hyperparameter tuning plays a critical role in prediction accuracy; however, the large number of coupled parameters in deep learning models renders manual trial-and-error strategies inefficient. Consequently, intelligent optimization algorithms have been widely introduced to improve search efficiency and enhance model performance,^31^ including WOA for joint hyperparameter tuning in CNN-LSTM attention models,^32^ SSA-based optimization in integrated SVR frameworks,^33^ and AMPSO for CNN hyperparameter tuning in gas-related applications.^34^ Inspired by projection iteration principles, Yu et al. proposed the Projection Iterative Optimization Algorithm (PIMO), which introduces four operators to guide population evolution and accelerate convergence. Given the large and highly interdependent hyperparameter space of the proposed PatchTST-PSA model, this study adopts PIMO to identify an effective hyperparameter configuration and further improve prediction performance.

### Research gap and novelty

In SRU sulfur-gas concentration forecasting, the coexistence of strong non-stationarity, multi-scale temporal dynamics, and high-frequency perturbations makes it difficult for a single modeling technique to achieve both noise robustness and accurate long-term dependency learning. Signal decomposition methods are effective in separating heterogeneous temporal structures but cannot directly provide predictive capability, whereas forecasting models trained on raw sequences are often sensitive to noise contamination and cross-scale interference. Accordingly, an effective SRU forecasting framework should integrate multi-stage decomposition with a dedicated prediction model, such that noise-suppressed and structurally interpretable sub-sequences can be constructed for subsequent temporal learning.

Based on the above review, several critical research gaps can be identified in sulfur gas concentration prediction for chemical processes, particularly in SRU applications.

(a) Most existing approaches directly model raw sulfur gas concentration sequences without explicitly disentangling non-stationary components and high-frequency disturbances. Such strategies limit the ability of prediction models to capture fine-grained dynamic behaviors, especially under highly volatile operating conditions, where rapid fluctuations and weak-structured signals are easily obscured by noise.
(b) Although deep learning models exhibit strong nonlinear modeling capability, their performance is often sensitive to hyperparameter configuration. Most existing approaches primarily rely on empirical tuning or grid-based search strategies, which tend to incur high computational cost, slow convergence, and limited efficiency in exploring complex parameter spaces. These limitations constrain the effective deployment of deep learning models in complex industrial environments.
(c) In sulfur gas prediction tasks, multi-source information is commonly fused as a unified input without sufficiently accounting for the heterogeneity across different modalities or the potential cross-scale coupling between process variables and environmental factors. As a result, the underlying interaction mechanisms among multi-source features are inadequately characterized, which reduces prediction accuracy and robustness.

This article proposes a hybrid forecasting framework, termed PatchTST-PSA, which integrates two-stage signal decomposition, entropy-guided reconstruction, and intelligent hyperparameter optimization for sulfur-gas concentration prediction in SRU processes. Specifically, the original gas concentration sequence is first decomposed using VMD to extract dominant modal components and a residual term, after which CEEMDAN is applied to the VMD residual to further disentangle weak-structured components and high-frequency disturbances; this two-stage decomposition strategy is referred to as VDES and serves as the front-end module of the proposed framework. Subsequently, all extracted intrinsic mode functions (IMFs) are evaluated using sample entropy, based on which entropy-guided aggregation and selection are performed to reduce the number of modeling sub-sequences while preserving informative dynamic characteristics. Finally, each reconstructed sub-sequence is modeled using the PatchTST-PSA predictor to capture temporal dependencies and interactions with environmental factors, while the key hyperparameters of PatchTST-PSA are optimized using PIMO to further improve overall prediction performance.

The main contributions of this study are summarized as follows.

(1) A sparse probabilistic self-attention mechanism (ProbSparse) is incorporated into the PatchTST backbone, resulting in the proposed PatchTST-PSA model, to mitigate redundant computation in long-sequence forecasting. By selectively emphasizing dominant queries, this design reduces computational and memory overhead while preserving the capability to model cross-patch long-range dependencies, thereby improving robustness for sulfur-gas concentration forecasting in noisy SRU environments.
(2) A two-stage decomposition strategy (VDES) is developed for SRU sulfur-gas signals. Specifically, VMD is employed to extract dominant modal structures, while CEEMDAN is introduced to further decompose the VMD residual so as to characterize high-frequency and non-stationary disturbances. In addition, sample entropy is utilized to quantify component complexity and guide aggregation and reconstruction, thereby reducing modeling complexity and mitigating error accumulation during reconstruction.
(3) The projection iterative optimization algorithm (PIMO) is used to optimize key hyperparameters of the PatchTST-PSA model. By leveraging projection-driven population updates and convergence control, PIMO enhances search efficiency and improves predictive performance and stability compared with manual tuning or basic search strategies.
(4) The proposed framework is validated using an Italian SRU dataset involving two representative sulfur-gas variables (H_2_S and SO_2_). Experimental results demonstrate consistent performance improvements over benchmark models across multiple evaluation metrics, indicating enhanced prediction accuracy and robustness under complex operating conditions and supporting its practical applicability in industrial scenarios.

The remainder of this article is organized as follows: the method details section presents the key methodologies, the overall framework of the proposed model, and the description of the SRU dataset. The results section provides a comprehensive analysis of the experimental results and compares them with several benchmark models. The discussion section discusses the findings, while the limitations of the study section outlines the limitations of the study.

## Results

### Hydrogen sulfide concentration sequence prediction results

#### Melting experiment

**Figure 1:**
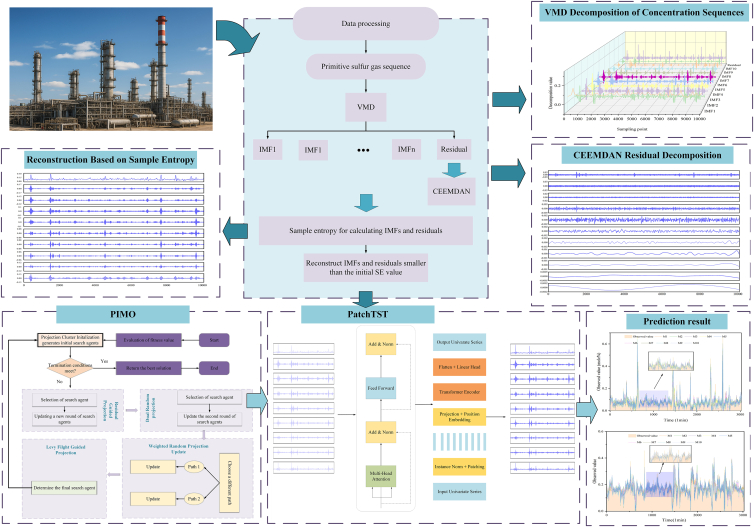
Framework of sulfur gas forecasting via VMD-CEEMDAN decomposition, entropy-based fusion, and PatchTST Industrial SRU sulfur-gas sequences are first preprocessed and decomposed by VMD and CEEMDAN. Next, sample-entropy-guided evaluation is used to screen and reconstruct informative components. Finally, PIMO performs global hyperparameter optimization for the PatchTST backbone to output multi-step predictions.

**Figure 2:**
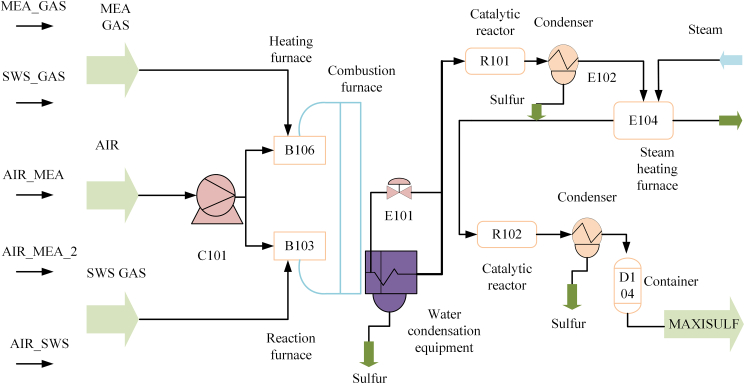
Sulfur recovery process diagram Acid-gas feeds (MEA_GAS and SWS_GAS) are mixed with air and processed through the main SRU train, including burner/thermal section units (B106, B103), catalytic reactors (R101, R102), and downstream heat-exchange/condensation and separation equipment (E104 and D104). The monitored outlet H_2_S and SO_2_ concentrations at the final stage are used as the target time series for model development and evaluation.

**Figure 3:**
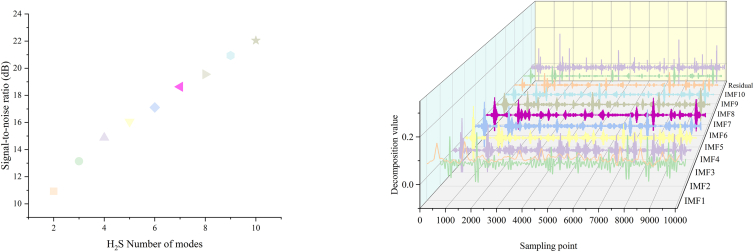
H_2_S decomposition results (Left) Signal-to-noise ratio (SNR) analysis used to determine the VMD mode number *K*. *K* = 10 (marked with a star) is selected to balance decomposition granularity and computational efficiency. (Right) 3D visualization of VMD outputs. The H_2_S sequence is decomposed into 10 modes (Mode 1–Mode 10) and a residual component, separating multi-scale temporal features for subsequent modeling.

**Figure 4:**
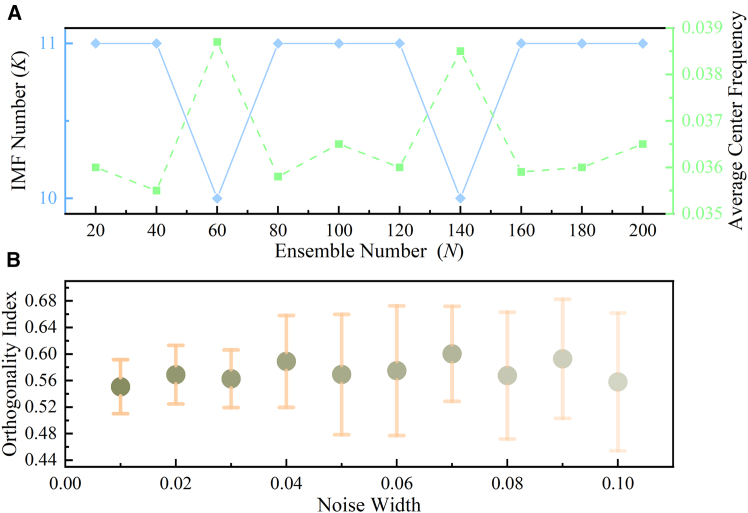
Sensitivity analysis of CEEMDAN parameters (A) Effect of ensemble size N (with *ϵ* = 0.05) on the number of IMFs *K* and the average center frequency *f* (B) Effect of noise amplitude *ϵ* (with *N* = 100) on the orthogonality index (OI). Data are shown as mean ± SD over *M* = 10 repeated runs.

**Figure 5:**
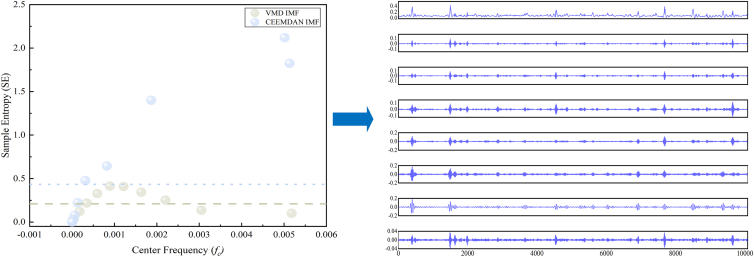
H_2_S sequence sample entropy aggregation Left: Scatterplot of sample entropy (SE) versus center frequency (*fc*) for IMFs obtained from VMD and CEEMDAN. Horizontal dashed lines denote the SE thresholds used to select low-complexity components for reconstruction. Right: Reconstructed sequences formed by aggregating the selected IMFs (SE below the corresponding thresholds), yielding denoised multi-scale features for subsequent forecasting.

To enhance the clarity and comparability of the experimental results, the ten prediction models are denoted as M1–M10, with their specific model compositions and signal processing strategies summarized in Table S1. The overall framework and detailed sulfur recovery process diagram are illustrated in Figures 1 and 2. The key operational input variables of the industrial SRU process and the target gas concentrations are detailed in Table 1, while the sample entropy values used for two-tier component screening are listed in Table 2. The decomposition results and parameter sensitivity analysis are provided in Figures 3, 4, and 5. The quantitative prediction performance of all models on the H_2_S dataset is reported in Table 3 and Figure S7, using four evaluation metrics, namely RMSE, MAE, MAPE, and R^2^, which collectively characterize prediction accuracy, stability, and goodness-of-fit.

**Table 1:** Input and output variables of the industrial SRU process key operational inputs

No.	Variables description
*x*_*1*_	MEA airflow
*x*_*2*_	SWS airflow
*x*_*3*_	Airflow in MEA zone
*x*_*4*_	Airflow in SWS zone
*x*_*5*_	Secondary airflow
*y*_*1*_	Concentration of H_2_S
*y*_*2*_	Concentration of SO_2_

Italic symbols (*x*_*1*_–*x*_*5*_) and target gas concentrations (*y*_*1*_–*y*_*2*_) denote the specific variables used in the forecasting models.

**Table 2:** Sample entropy of each component of the H_2_S concentration series

VMD Component	SE	CEEMDAN	SE
IMF_raw_	0.2104	IMF_res_	0.4332
IMF1	0.1009	IMF1	1.8249
IMF2	0.1366	IMF2	2.1199
IMF3	0.2520	IMF3	1.4007
IMF4	0.3437	IMF4	0.6452
IMF5	0.4089	IMF5	0.4772
IMF6	0.4139	IMF6	0.2230
IMF7	0.3281	IMF7	0.0777
IMF8	0.2185	IMF8	0.0311
IMF9	0.1222	IMF9	0.0106
IFM10	0.0384	IMF10	0.0032
		IFM11	9.8808E-4

SE values used for two-tier component screening: low-SE components are aggregated for forecasting, while high-SE CEEMDAN components are excluded as noise.

**Table 3:** Comparison of H_2_S concentration predictions

Model	Model ID	RMSE (mole%)	MAE (mole%)	R^2^	MAPE (%)
BP	M1	0.0379	0.0231	0.5697	33.04%
GRU	M2	0.0355	0.0226	0.6224	31.09%
LSTM	M3	0.0324	0.0185	0.6844	30.06%
Transformer	M4	0.0310	0.0206	0.7109	29.48%
Informer	M5	0.0285	0.0181	0.7565	29.99%
PatchTST	M6	0.0256	0.0177	0.8028	29.97%
PatchTST-PSA	M7	0.0225	0.0156	0.8484	26.15%
VMD-PatchTST-PSA	M8	0.0199	0.0143	0.881	23.47%
VDES-PatchTST-PSA	M9	0.0178	0.0131	0.9049	19.38%
VDES-PIMO-PatchTST-PSA	M10	**0.0138**	**0.0104**	**0.9429**	**17.19%**

Performance of the proposed VDES-PIMO-PatchTST-PSA model (M10) versus baseline and ablation models (M1–M9) evaluated by RMSE, MAE, R^2^, and MAPE. Best results are highlighted in bold.

**Figure 6:**
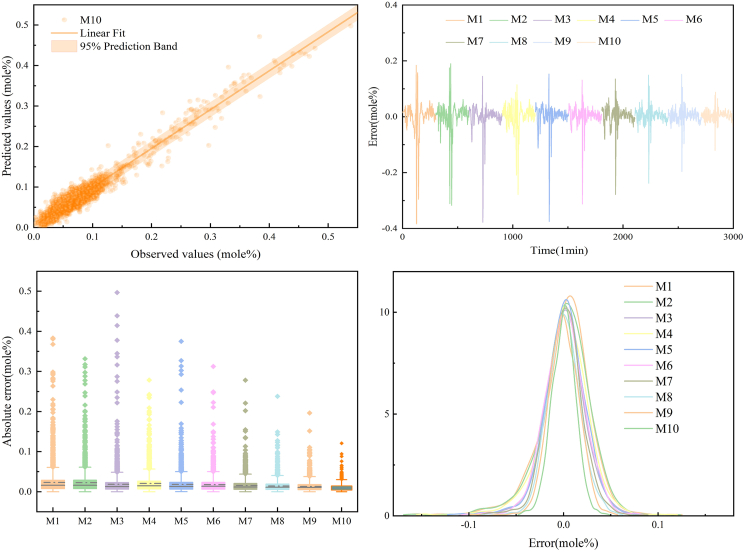
Regression fitting and error analysis of different models on the H_2_S dataset (Top left) Regression between observed and predicted H_2_S values for M10. The solid line denotes the linear fit, and the shaded region indicates the 95% prediction interval of the fitted regression. (Top right) Temporal prediction error sequences for models M1–M10. (Bottom left) Boxplots of absolute errors for different models (center line: median; box: interquartile range [IQR]; whiskers: 1.5×IQR; points: outliers). (Bottom right) Error distribution curves of prediction errors for models M1–M10.

A comprehensive visual comparison of representative models is presented in Figure 6, including regression plots, error time series, absolute error boxplots, and error distribution curves. As observed from the error time series, pronounced fluctuations occur within the 500–800 min interval, highlighting the models’ responsiveness to abrupt variations and abnormal disturbances. The error density plots further reveal notable differences in error concentration and distribution normality among the competing approaches, providing insight into their robustness. Moreover, Figure S8 compares the predicted and observed H_2_S concentrations and includes a locally enlarged view focusing on peak segments, from which the VDES-PIMO-PatchTST-PSA model exhibits superior tracking accuracy and robustness during periods of rapid change.

Based on the quantitative metrics and visualization results, a systematic ablation analysis is conducted to evaluate the contribution of each component in the proposed framework.

(a) Compared with conventional baseline models (BP, GRU, LSTM, Transformer, and Informer), the PatchTST model demonstrates consistently superior forecasting performance, indicating its effectiveness in capturing long-range temporal dependencies in H_2_S concentration series.
(b) By incorporating probabilistic sparse attention, PatchTST-PSA further improves prediction accuracy, with the RMSE and MAE decreasing from 0.0256 to 0.0225 and from 0.0177 to 0.0156, respectively, while the MAPE is reduced from 29.97% to 26.15% and the R^2^ increases from 0.8028 to 0.8484.
(c) The introduction of variational mode decomposition leads to additional performance gains. Compared with PatchTST-PSA, the VMD-PatchTST-PSA model reduces the RMSE and MAE from 0.0225 to 0.0199 and from 0.0156 to 0.0143, respectively, while improving the R^2^ from 0.8484 to 0.8818, confirming the effectiveness of multi-scale decomposition in mitigating non-stationarity.
(d) To further capture high-frequency perturbations, CEEMDAN-based secondary decomposition and sample entropy-guided reconstruction are employed, resulting in the VDES-PatchTST-PSA model. Compared with VMD-PatchTST-PSA, the RMSE, MAE, and MAPE are reduced from 0.0199 to 0.0178, from 0.0143 to 0.0131, and from 23.47% to 19.38%, respectively, while the R^2^ increases from 0.8818 to 0.9049, demonstrating enhanced robustness and stability.
(e) Finally, the effectiveness of PIMO-based hyperparameter optimization is validated by comparing VDES-PIMO-PatchTST-PSA with VDES-PatchTST-PSA. After optimization, the RMSE further decreases from 0.0178 to 0.0138, the MAE from 0.0131 to 0.0104, and the MAPE from 19.38% to 17.19%, while the R^2^ increases from 0.9049 to 0.9429, indicating that PIMO substantially enhances predictive accuracy and model robustness.

#### Threshold sensitivity analysis

To assess the robustness of the entropy-based reconstruction strategy, both entropy thresholds were jointly scaled by a factor γ ∈ {0.8, 0.9, 1.0, 1.1, 1.2}, while all other model components and hyperparameters were kept unchanged. As shown in Table S2, when γ varies from 0.8 to 1.0, the reconstructed subsequence structure remains unchanged, with eight subsequences retained for modeling, and identical prediction performance is achieved. This indicates the existence of a stable threshold interval, within which moderate perturbations of the entropy thresholds do not affect either the reconstruction topology or the forecasting accuracy.

When γ increases to 1.1 and 1.2, the entropy thresholds become more permissive, leading to stronger aggregation of decomposed components and a reduction in the number of reconstructed subsequences. Consequently, a gradual degradation in prediction performance is observed, which can be attributed to reduced flexibility in representing fine-scale temporal variations and partial loss of informative high-frequency dynamics. Overall, these results demonstrate that the proposed entropy-guided reconstruction is robust to threshold selection within a reasonable range, while excessively large thresholds may cause information loss. Therefore, the adopted threshold setting (γ = 1.0) achieves a favorable balance between noise suppression and information preservation.

#### Computational cost analysis

All experiments were conducted on a workstation equipped with an NVIDIA RTX 4090 GPU (24 GB memory), an Intel Xeon Platinum 8352 V CPU, and 120 GB system RAM, running Ubuntu 20.04. The models were implemented using PyTorch 1.10.0 with Python 3.8 and CUDA 11.3, and all experiments were performed using single-precision floating-point arithmetic.

As shown in Table S3, the baseline PatchTST has the lowest computational cost as a single-stage model, processing only the original sequence. Sparse probabilistic attention slightly increases training time while reducing peak GPU memory usage, offering a favorable trade-off between efficiency and resource consumption. With multi-stage decomposition (e.g., VDES), complexity increases with the number of reconstructed subsequences. However, even with 8 subsequences in our model, per-epoch training time remains efficient, and memory consumption is controlled through modular sub-model reuse.

Notably, the proposed VDES-PIMO-PatchTST-PSA model demonstrates superior computational efficiency despite its multi-stage architecture and the incorporation of 8 subsequences. Its inference latency remains within the millisecond range, which is competitive with other PatchTST-based variants. Overall, the computational overhead is well-balanced with the significant gains in prediction accuracy and robustness, justifying its application in SRU sulfur-gas concentration forecasting.

### Sulfur dioxide prediction results and generalization analysis

To further validate the robustness of the VDES-PIMO-PatchTST-PSA framework, we extended the evaluation to SO_2_ concentration forecasting. Although SO_2_ exhibits similarly rapid dynamics to H_2_S, its distinct volatility patterns and concentration ranges provide a more stringent test of cross-target generalization. As summarized in Table S4 and visualized in Figures S9, S10, and 7, M10 consistently achieves the best overall accuracy, indicating that the observed gains are attributable to the proposed design rather than chance.

The error time series in Figure S10 further highlights M10’s stability under abrupt disturbances. In the highly turbulent interval between 2500 and 2800 min, M10 shows smaller error excursions than the baselines (M6–M9). The corresponding absolute-error boxplots corroborate this behavior, where M10 presents a more compact distribution with fewer extreme outliers. Such suppression of peak errors is particularly important for practical SRU monitoring, where large deviations can compromise operational safety.

Consistent with these observations, the regression and residual analyses (Figures S10 and 7) show that M10 maintains high agreement with measurements across the testing range. The predicted-observed scatter is closely aligned with the fitted trend, and the 95% prediction bands remain relatively narrow, reflecting tighter dispersion around the fit. Quantitatively, M10 achieves an R^2^ of 0.9228 and an RMSE of 0.0152, corresponding to a 36.40% RMSE reduction relative to PatchTST (M6). Overall, these results support that combining VDES, PIMO, and PSA improves robustness and accuracy for SO_2_ forecasting and transfers effectively across gas targets.

**Figure 7:**
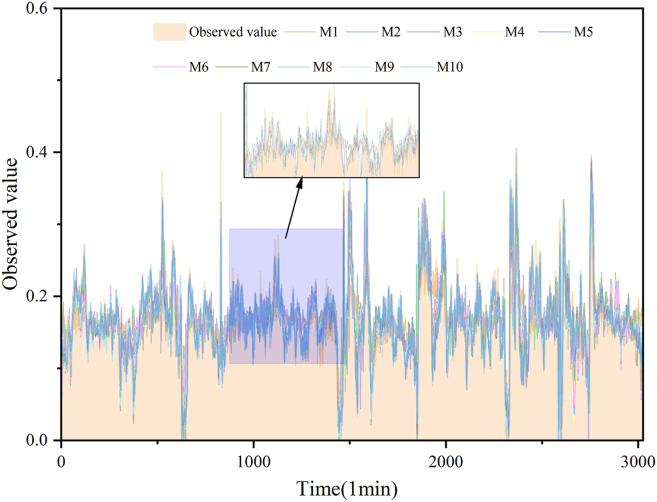
Comparison of predicted and observed SO2 concentration curves over time Time-series comparison between the observed SO_2_ concentration (orange shaded curve) and predictions from models M1–M10. The inset zooms in on the 900–1500 min interval to facilitate the visual inspection of short-term fluctuations and rapid changes, where M10 shows closer agreement with the observations.

### Percentage of improvement in prediction results by different methods

This study quantifies improvements in predictive performance across various models by calculating the percentage improvement in computational models. The method involves comparing the prediction results of the original model with those of the enhanced model, calculating the differences in each performance metric, and converting these differences into percentage values. This approach facilitates a clear and standardized assessment of model improvement effects, providing an objective basis for comparing various models. The proposed model represents an updated approach, while the other models refer to the original models. The calculation formula is as follows:

(Equation 1) $$P_{\text{RMSE}}=\frac{{\text{RMSE}}_{\text{original}}-{\text{RMSE}}_{\text{new}}}{{\text{RMSE}}_{\text{original}}}\times 100\%$$

(Equation 2) $$P_{\text{MAE}}=\frac{{\text{MAE}}_{\text{original}}-{\text{MAE}}_{\text{new}}}{{\text{MAE}}_{\text{original}}}\times 100\%$$

(Equation 3) $$P_{\text{MAPE}}=\frac{{\text{MAPE}}_{\text{original}}-{\text{MAPE}}_{\text{new}}}{{\text{MAPE}}_{\text{original}}}\times 100\%$$

The results are shown in Table S5, from which the following conclusions can be drawn:

In the H_2_S prediction task, the proposed model exhibits significant improvements across four metrics when compared to the baseline model. Specifically, the RMSE, MAE, and MAPE of VDES-PIMO-PatchTST-PSA decreased by 46.09%, 41.24%, and 42.64%, respectively, in relation to PatchTST. When compared to PatchTST-PSA, the RMSE decreased by 38.67%, the MAE by 33.33%, and the MAPE by 34.26%. These substantial performance gains can be attributed to the following factors.

(i) VDES explicitly decouples dominant-scale trends from high-frequency and non-stationary disturbances. It suppresses random noise while enhancing the signal-to-noise ratio through sample entropy-driven reconstruction.
(ii) PatchTST-PSA employs a sparse probabilistic self-attention mechanism that focuses on dominant queries. This approach preserves critical long-range dependencies while simultaneously reducing redundant attention overhead and mitigating noise amplification.
(iii) PIMO employs projection-iterative joint optimization to fine-tune key hyperparameters, including learning rate, batch size, patch length, and hidden dimension. This approach facilitates an improved balance between global exploration and local refinement while effectively mitigating premature convergence.

Overall, the aforementioned mechanism significantly enhances the model’s accuracy, stability, and generalization capability under complex industrial time-series conditions.

## Discussion

The SRU process exhibits pronounced nonlinearity and time-varying dynamics due to the coupled effects of temperature, pressure, and sulfur-gas concentrations, which makes accurate forecasting challenging. The proposed VDES-PIMO-PatchTST-PSA framework addresses this by integrating multi-scale decomposition, attention optimization, and global hyperparameter tuning. Across both the H2S and SO2 datasets, the framework consistently outperforms BP, GRU, vLSTM, Transformer, and Informer in terms of RMSE, MAE, MAPE, and R2, indicating improved accuracy and generalization.

The performance gain is largely attributable to three design choices. First, PatchTST benefits from patch-based embedding, which preserves local temporal patterns while enabling efficient long-sequence modeling, leading to better representation of SRU multi-scale dynamics. Second, PSA further improves PatchTST by emphasizing informative token interactions and reducing redundant attention computation, which helps capture critical long-range dependencies. Third, the VMD-CEEMDAN dual-stage decomposition, together with sample-entropy-guided reconstruction, enhances scale separation and retains informative non-stationary/high-frequency components, providing more structured inputs for prediction. Finally, PIMO-based global optimization mitigates manual tuning and local optima, improving robustness under highly fluctuating operating conditions.

### Limitations of the study

Despite the performance gains of the VDES-PIMO-PatchTST-PSA model, certain limitations remain. First, the residual-oriented two-stage decomposition based on VMD and CEEMDAN is applied only to the output sequences of H_2_S and SO_2_, while input variables are not yet processed in a multiscale manner. Future work may extend the framework by performing synchronous multiscale decomposition on input features to better capture underlying dynamic structures. Second, although multi-stage decomposition and intelligent hyperparameter optimization improve prediction performance, they also increase computational complexity and training cost. Future research may focus on enhancing efficiency through algorithmic simplification or model compression to reduce latency in industrial deployments.

## Resource availability

### Lead contact

Further information and requests for resources and data should be directed to and will be fulfilled by the lead contact, Binglin Lu (lubinglin@sdut.edu.cn).

### Materials availability

This study did not generate new unique materials.

### Data and code availability

• The benchmark industrial sulfur gas concentration dataset used for model training and testing has been publicly deposited in Mendeley Data: https://doi.org/10.17632/6p865gztg6.1.
• The core code framework and algorithms for constructing the hybrid multiscale forecasting model have been publicly deposited in Zenodo: https://doi.org/10.5281/zenodo.18357461.
• Any additional information required to reanalyze the data reported in this article is available from the lead contact upon request.

## Acknowledgments

This work was supported by the 10.13039/501100007129Shandong Provincial Natural Science Foundation (Grant No. ZR2022MF344) and the Shandong Provincial Key Research and Development Program (Grant No. 2023CXPT089).

## Author contributions

Author Contributions W.S. and L.L. performed the experiments and analyzed the data. B.L. conceptualized the research, supervised the work, and served as the lead contact. L.J. and J.Z. contributed to methodology and provided technical resources. All authors discussed the results and contributed to the final article.

## Declaration of interests

The authors declare no competing interests.

## STAR★Methods

### Key resources table

**Table:** 

REAGENT or RESOURCE	SOURCE	IDENTIFIER
**Deposited data**		

Industrial SRU operational dataset	This paper; Italian Refinery	Mendeley Data: https://doi.org/10.17632/6p865gztg6.1

**Software and algorithms**		

Python (v.3.8)	Python Software Foundation	https://www.python.org/
PyTorch (v.1.10.0)	PyTorch	https://pytorch.org/
Variational Mode Decomposition	Liu et al.^35^	DOI: https://doi.org/10.1016/j.energy.2024.130726
CEEMDAN	Torres et al.^36^	DOI: https://doi.org/10.1109/ICASSP.2011.5947265
PatchTST	Nie et al.^21^	https://github.com/yuqinie98/PatchTST
Optimized PatchTST and VMD-CEEMDAN scripts	This paper	Zenodo: https://doi.org/10.5281/zenodo.18357461
PIMO Hyperparameter Optimizer	This paper	DOI: https://doi.org/10.5281/zenodo.18357461

**Other**		

NVIDIA RTX 4090 GPU	NVIDIA	https://www.nvidia.com/
Ubuntu 20.04 LTS	Canonical	https://ubuntu.com/

### Experimental model and study participant details

This study does not involve any experimental models using organisms (animals, plants, or microbes), human participants, or cell lines. The research is based on historical operational data obtained from an industrial Sulfur Recovery Unit (SRU). Therefore, information regarding species, strain, genotype, age, sex, and maintenance/care is not applicable. For human subjects-related reporting, as no human participants were involved, sex/gender association analysis and institutional oversight were not required.

### Method details

#### Variational mode decomposition

Variational Mode Decomposition (VMD)^35^ is a signal decomposition method grounded in a variational framework, characterized by exceptional theoretical controllability and concentration in the frequency domain, which renders it particularly suitable for the processing of non-stationary signals. Using a variational formulation, VMD separates the input signal into a set of intrinsic mode functions with limited bandwidth. Each mode reflects a characteristic fluctuation in a defined frequency range, thereby facilitating efficient feature extraction across scales. In comparison to traditional methods, VMD exhibits enhanced decomposition accuracy and stability.

Assuming the sulfur gas concentration sequence is *x(t)*, the VMD decomposition process is as follows.

Step 1: The objective of VMD is to decompose the signal *x(t)* into *K* modal components {*u*_*k*_(t)}. The bandwidth of each mode is expressed through the frequency shift form of its Hilbert analytical signal and measured using the squared norm. The overall optimization problem is as follows:

(Equation 4) $$\min_{\left\{u_{k}\right\},\left\{\omega_{k}\right\}}\left\{\sum_{k=1}^{K}\partial_{t}{\left[\left(\delta \left(t\right)+\frac{j}{\pi t}\right)*u_{k}\left(t\right)\cdot e^{-j\omega_{k}t}\right]}_{2}^{2}\right\}$$

Here, *w*_*k*_ denotes the center frequency of the *k*th mode, and ∗ represents the convolution operation. To ensure signal reconstructability, the following constraint is imposed:

(Equation 5) $$\sum_{k=1}^{K}u_{k}\left(t\right)=x\left(t\right)$$

Step 2: To address the aforementioned constrained optimization problem, a Lagrange multiplier *λ(t)* and a penalty factor *α* are introduced to construct the augmented Lagrange function as follows:

(Equation 6) $$L=\alpha \sum_{k=1}^{K}\partial_{t}{\left[\left(\delta \left(t\right)+\frac{j}{\pi t}\right)*u_{k}\left(t\right)\cdot e^{-j\omega_{k}t}\right]}_{2}^{2}+x\left(t\right)-\sum_{k=1}^{K}u_{k}{\left(t\right)}_{2}^{2}+\lambda \left(t\right),x\left(t\right)-\sum_{k=1}^{K}u_{k}\left(t\right)$$

The first term measures bandwidth, the second term represents the penalty for constraint violations, and the third term is the Lagrangian penalty for reconstruction error.

Step 3: In the frequency domain, the Alternating Direction Method of Multipliers (ADMM) is employed to iteratively update each variable. First, the spectral representation of the kth modal component is updated:

(Equation 7) $${\hat{u}}_{k}^{n+1}\left(\omega \right)=\frac{\hat{x}\left(\omega \right)-\sum_{i\neq k}{\hat{u}}_{i}\left(\omega \right)+\frac{1}{2}\hat{\lambda }\left(\omega \right)}{1+2\alpha {\left(\omega -\omega_{k}\right)}^{2}}$$

Step 4: Update the modal center frequency *w*_*k*_ by utilizing the spectral energy distribution of the current mode to update its center frequency:

(Equation 8) $$\omega_{k}^{n+1}=\frac{\int \omega \cdot {\left|{\hat{u}}_{k}\left(\omega \right)\right|}^{2}d\omega }{\int {\left|{\hat{u}}_{k}\left(\omega \right)\right|}^{2}d\omega }$$

Step 5: Update the Lagrange multiplier *λ(t)* to strengthen the signal reconstruction consistency constraint:

(Equation 9) $${\hat{\lambda }}^{n+1}\left(\omega \right)={\hat{\lambda }}^{n}\left(\omega \right)+\tau \left(\hat{x}\left(\omega \right)-\sum_{k=1}^{K}{\hat{u}}_{k}\left(\omega \right)\right)$$

Here, *τ* is the step size coefficient, used to adjust the update rate.

Step 6: Repeat steps 3 to 5 until all modes satisfy the following convergence criteria:

(Equation 10) $$\frac{∥u_{k}^{n+1}-u_{k}^{n}{∥}_{2}}{∥u_{k}^{n}{∥}_{2}}<\epsilon ,\forall k$$

Ultimately, the signal *x(t)* is decomposed into the sum of *K* eigenmode functions and a residual term:

(Equation 11) $$x\left(t\right)=\sum_{k=1}^{K}u_{k}\left(t\right)$$

#### Sample entropy

Sample entropy (SE)^37^ is a nonlinear dynamic metric used to measure the complexity and uncertainty of time series, as proposed by Richman and Moorman in 2000. Developed from approximate entropy, sample entropy mitigates biases introduced by self-matching, providing enhanced robustness and accuracy. In this paper, we calculate the SE values for each component based on sample entropy through the following steps.

Step 1: Reconstruct the sequence {*x*_*1*_*, x*_*2*_*,⋯x*_*i+m-1*_} of length *N* into a set of m-dimensional vectors, i.e., construct *N-m* + *1* embedding vectors:

(Equation 12) $$X_{i}^{\left(m\right)}=\left[x_{i},x_{i+1},\ldots ,x_{i+m-1}\right],i=1,2,\ldots ,N-m+1$$

Step 2: For each $X_{i}^{\left(m\right)}$, compute the maximum distance to other vectors $x_{j}^{\left(m\right)}$ (where *j*≠*i*):

(Equation 13) $$d\left(X_{i}^{\left(m\right)},X_{j}^{\left(m\right)}\right)=\max_{0\leq k<m}\left|x_{i+k}-x_{j+k}\right|$$

Count the number of matching pairs *B*_*i*_ that satisfy *d* < *r*, and calculate the average:

(Equation 14) $$B=\frac{1}{N-m+1}\sum_{i=1}^{N-m+1}\frac{B_{i}}{N-m}$$

Step 3: Extend the embedding dimension to m+1, repeat Steps 1 and 2, and obtain the new average matching ratio *A.*
Step 4: Sample entropy is ultimately defined as:

(Equation 15) $$\text{SE}\left(m,r,N\right)=-\ln\left(\frac{A}{B}\right)$$

Here, $\frac{A}{B}$ denotes the change in matching probability from dimension m to m+1.

#### Complete ensemble empirical mode decomposition with adaptive noise

CEEMDAN (Complete Ensemble Empirical Mode Decomposition with Adaptive Noise) represents a significant advancement over the EMD (Empirical Mode Decomposition) series of algorithms, effectively addressing the issues of mode overlap and reconstruction error that are prevalent in EMD and its earlier variants.

Huang et al.^38^ introduced EMD in 1998 as an adaptive technique for breaking nonlinear, non-stationary signals into intrinsic mode functions (IMFs). Nonetheless, EMD is hindered by issues related to modal overlap. To address this issue, Wu and Huang^39^ introduced Ensemble Empirical Mode Decomposition (EEMD). This method involves adding distinct white noise to the original signal multiple times, performing EMD on each instance, and subsequently averaging all Intrinsic Mode Function (IMF) components. This approach effectively mitigates the modal overlap problem inherent in EMD. However, the EEMD method is plagued by significant reconstruction errors. To address this issue, Torres et al.^36^ introduced the CEEMDAN in 2011. This method incorporates a residual re-noising mechanism, allowing each Intrinsic Mode Function (IMF) component to be extracted from the residuals left by the preceding IMF, rather than relying on repeated decomposition of the entire signal. Consequently, this approach retains the advantages of EEMD while effectively mitigating its reconstruction error problems.

Let the original time series be denoted as *x(t)*. The objective of CEEMDAN is to decompose it into several intrinsic modal functions (IMFs) and a residual term *r*_*k*_*(t)*, following these steps.

Step 1: Add white noise *w*_*i*_*(t)* from different realizations to the original signal *x(t)* to form noisy samples:

(Equation 16) $$x_{i}\left(t\right)=x\left(t\right)+\varepsilon_{0}w_{i}\left(t\right),i=1,2,\ldots ,N$$

Among these, *ε*_*0*_ represents the initial noise amplitude, and *N* denotes the number of integration cycles.

Step 2: Perform EMD on each *x*_*i*_*(t)*, extract its first eigenmode function, and compute the average:

(Equation 17) $${\text{IMF}}_{1}\left(t\right)=\frac{1}{N}\sum_{i=1}^{N}h_{1}^{\left(i\right)}\left(t\right)$$

Step 3: Remove the first mode from the original signal to obtain the residual signal:

(Equation 18) $$r_{1}\left(t\right)=x\left(t\right)-{\text{IMF}}_{1}\left(t\right)$$

Step 4: Add a new white noise sequence to the residual *r*_*1*_*(t)* to construct the noisy residual:

(Equation 19) $$r_{1}^{\left(i\right)}\left(t\right)=r_{1}\left(t\right)+\varepsilon_{1}w_{i}\left(t\right)$$

Then perform EMD on each signal, extracting the corresponding IMF for each, and calculate its mean as the second modal component:

(Equation 20) $${\text{IMF}}_{2}\left(t\right)=\frac{1}{N}\sum_{i=1}^{N}h_{2}^{\left(i\right)}\left(t\right)$$

Step 5: For each new residual *r*_*k*_*(t)* = *r*_*k-1*_*(t)-IMF*_*k*_*(t)*, repeat Step 4 to continue extracting new IMFs until the termination criterion is satisfied:

(Equation 21) $$r_{k}\left(t\right)=r_{k-1}\left(t\right)-{\text{IMF}}_{k}\left(t\right)$$

Step 6: Ultimately, the original signal is expressed as the sum of several intrinsic modal functions and residual terms:

(Equation 22) $$x\left(t\right)=\sum_{k=1}^{K}{\text{IMF}}_{k}\left(t\right)+r_{K}\left(t\right)$$

#### Projection-Iterative-Methods-based optimizer

PIMO (Projection-Iterative-Methods-based Optimizer) represents a class of meta-heuristic algorithms that leverage projection–iteration principles, specifically developed for continuous optimization and feature selection problems. The algorithm employs four complementary operators: Residual-Guided Projection (RGP), Dual Random Projection (DRP), Weighted Random Projection Update (WRPU), and Lévy Flight-Guided Projection (LFGP) for operator scheduling. By maintaining feasible domain projections, it achieves a dynamic equilibrium between global exploration and local refinement. Combined with Kaczmarz-like updates and principles of Stochastic Gradient Descent (SGD), this approach mitigates premature convergence. In contrast to conventional swarm-based methods such as Particle Swarm Optimization (PSO), Whale Optimization Algorithm (WOA), and Differential Evolution (DE), PIMO differentiates itself through explicit directional projection and a one-step processing of feasibility constraints. This characteristic endows it with enhanced stability and transferability in hybrid hyperparameter optimization and engineering optimization tasks that involve bounded or discrete mappings.

#### Initialization of the projection swarm

To initiate the optimization process of PIMO, a set of *N* projection search individuals with variable dimensions *D* is randomly generated within the feasible region. The initial coordinate of the *i-th* individual along dimension *j* is:

(Equation 23) $$X_{ij}=r_{0}\left({UB}_{j}-{LB}_{j}\right)+{LB}_{j}$$

Here, *X*_*ij*_ denotes the coordinate of the *i-th* (projection-search) individual in dimension *j*; *r*_*0*_*∼U (0,1)*; and *UB*_*j*_, *LB*_*j*_ are the upper and lower bounds of dimension *j*. For components exceeding their defined bounds, it is essential to perform feasible region projection. Additionally, for variables that are sensitive to log-scale transformations, sampling and projecting should be conducted within the log domain. In the case of discrete variables, a near-exhaustive mapping approach should be employed to guarantee type consistency and ensure reproducible initial diversity.

Here, *X*_*ij*_ denotes the coordinate of the *i-th* (projection-search) individual in dimension *j*; *r*_*0*_*∼U (0,1)*; and *UB*_*j*_, *LB*_*j*_ are the upper and lower bounds of dimension *j*.

#### Residual-guided projection

In the RGP stage, successive projections in a multi-dimensional space are performed to approximate the optimal solution. Figure S1 illustrates the workflow associated with this phase. This process integrates Kaczmarz iterative updates with the principles of Stochastic Gradient Descent (SGD) to offer trajectory guidance for searches within high-dimensional feasible regions. The algorithm randomly selects two candidate solutions, *X*_*v1*_ and *X*_*v2*_, which exhibit smaller residual fitness values, to serve as guiding surrogates. The projection gradient, G, is then calculated based on these selections.

(Equation 24) $$G_{1}=\frac{R\left(X_{v1}-X_{\text{best}}\right)+\left(1-R\right)\left(X_{v2}-X_{\text{best}}\right)}{2},if\;3r_{3}\geq 2r_{4}$$

Here, *r*_3_ and *r*_4_ are independent random numbers uniformly distributed in 0,1, used only in the condition 3*r*_3_ ≥ 2*r*_4_ to randomly select between the gradient-based update and the Jacobian-based update.

Otherwise, update G1 according to the alternative strategy. To enhance directional accuracy, introduce the approximate Jacobian matrix *J*, whose elements are:

(Equation 25) $$J_{ij}=\frac{\partial f_{j}}{\partial x_{i}}\;\approx \;\frac{f_{j}\left(x+\epsilon e_{i}\right)-f_{j}\left(x\right)}{\epsilon }$$

Where *e*_*i*_ is the standard basis and ϵ is the perturbation amplitude. The RGP update for the *t-th* iteration is:

(Equation 26) $$X_{n1}^{\text{proj}\left(t+1\right)}=\left\{ \begin{array}{l} X_{i}-\delta G_{1},3r_{3}\geq 2r_{4}\\ X_{i}+J\delta G_{1},\text{otherwise} \end{array}\right.$$

where the dynamic step size is

(Equation 27) $$\delta =\sin\left(\frac{\pi }{2}\left(1-{\left(\frac{2t}{T}\right)}^{5}\right)\right)$$

T denotes the maximum number of iterations. After updating, project back to the feasible region.

#### Dual random projection

DRP strengthens the algorithm’s global search by employing a two-tier randomization scheme. Figure S2 illustrates the process flow for this stage. It first samples two population indices, *v*_*3*_ and *v*_*4*_, to act as references for the projection update. Under the gradient-correction branch, the direction vector *g*_*1*_ is defined by subtracting the current solution from the incumbent optimum.

(Equation 28) $$g_{1}=\frac{R\left(X_{v3}-X_{\text{best}}\right)+\left(1-R\right)\left(X_{v4}-X_{\text{best}}\right)}{2}$$

Corresponding update

(Equation 29) $$X_{n2}^{\text{proj}\left(t+1\right)}=X_{i}-\delta g_{1}$$

If the Jacobian matrix update is selected, the update direction vector *g*_*2*_ is computed as:

(Equation 30) $$\begin{array}{l} g_{2}=\frac{R\times \left(X_{\nu 4}-X_{best}\right)+\left(1-R\right)\times \left(X_{\nu 3}-X_{best}\right)}{2}\\ X_{n2}^{proj\left(t+1\right)}=X_{i}+J\times \delta \times g_{2}^{T} \end{array}$$

Here, $X_{n3}^{proj\left(t+1\right)}$ denotes the solution following a single update of the DRP. By incorporating randomness in both the reference individual and the update direction, the DRP enhances process diversity, thereby facilitating the expansion of global search across various directions.

#### Weighted random projection update

The WRPU utilizes random weighting and adaptive adjustments to update positions, effectively guiding individuals toward optimal solutions within the solution space. This process incorporates factors *r*_*7*_ and *r*_*8*_ to determine the update weights, facilitating a biased progression toward the optimum.

(Equation 31) $$r_{7}=1+\text{rand},r_{8}=1+\text{rand}$$

Based on this, two complementary projection paths are constructed for position updates.

Path 1 employs a randomly weighted projection (bias-to-best), using a linear combination of the current solution *Xᵢ* and the optimal solution *X*_*best*_ as the update basis, along with an additional bias term that guides the solution toward the optimum:

(Equation 32) $$X_{n3}^{\text{proj}\left(t+1\right)}=r_{7}X_{i}+\left(1-R\right)X_{\text{best}}+r_{8}\left(X_{i}-X_{\text{best}}\right)$$

Where R ∈ [0,1] is an independent random variable used to adjust the attractiveness of the optimal solution and the diversity of the population.

Path 2 employs adaptive correction projection (history-corrected), utilizing the historical position $X_{n3}^{proj\left(t+1\right)}$ obtained during the RGP phase for directional correction.

(Equation 33) $$X_{n3}^{\text{proj}\left(t+1\right)}=X_{i}+a\left(X_{n1}^{\text{proj}\left(t+1\right)}-X_{\text{best}}\right),a=\left(1-\frac{t}{T}\right)\times \text{rand}$$

Where *a* is an adaptive step size that decays with iteration, and *t/T* controls the transition from exploration to exploitation.

#### Lévy-flight-guided projection

Through a long-tail migration–inspired mechanism, LFGP harmonizes broad exploratory moves with localized refinement. Its trigger probability is defined as follows:

(Equation 34) $$O=\frac{1}{2}\left(\tanh\left(9\frac{t}{T}-5\right)+1\right)$$

It increases monotonically with each iteration, facilitating exploration in the early stages and gradually transitioning to convergence in the later stages. The step size *z* is determined using the Mantegna scheme:

(Equation 35) $$z=\frac{u}{{\left|v\right|}^{1/\beta }}\times \tau$$

Where u,v∼N(0,1),β∈(0,2] controls the tail thickness, and *τ* is the scaling coefficient given by the gamma function. When the trigger condition is satisfied, the position is updated as:

(Equation 36) $$X_{n4}^{\text{proj}\left(t+1\right)}=r_{9}X_{\text{Elite}}+\left(1-r_{9}\right)z\times d\times \left(X_{\text{Elite}}-X_{j}\times \left(2^{t/T}\right)\right)$$

Where *X*_Elite_ represents the current elite solution, *X*_*j*_ denotes the reference individual for the current solution, *r*_*9*_ ∈ [0,1] is the random weight, and the dynamic weight $d=\text{rand}\times \left(1-{\left(\frac{t}{T}\right)}^{2}\right)$ is used for annealing the step size over time. After updating, projection onto the feasible region is performed to ensure that out-of-bounds components are constrained.

#### Patch-based time series transformer

PatchTST is a novel Transformer architecture proposed by Nie et al.^21^ This model introduces a patch segmentation mechanism that divides the original sequence into fixed-length sub-sequences. The model leverages self-attention to simultaneously capture local temporal features and global dependencies. The architecture comprises a patch-embedding module, a multi-head attention encoder, and a linear readout. The time series is partitioned into non-overlapping patches that act as input tokens, with no patch permutation applied so that temporal order is preserved.

#### Channel independence

In multivariate time series forecasting tasks, PatchTST employs a channel-independent modeling strategy. It decomposes the input multidimensional time series into *D* univariate sequences, performing patch segmentation and feature extraction on each sequence separately. Let the original input sequence be defined as follows:

(Equation 37) $$X\in R^{L\times D}$$

Here, *L* denotes the time step length, and *D* represents the number of dimensions. PatchTST divides it into *D* channels:

(Equation 38) $$X=\left\{x^{\left(1\right)},x^{\left(2\right)},\ldots ,x^{\left(D\right)}\right\},x^{\left(i\right)}\in R^{L}$$

Each sequence *x*^*(i)*^ from the various channels is partitioned into overlapping patches, which are processed as independent samples within the Transformer encoder. This decoupling approach allows each variable to independently capture temporal features within a unified structure, thereby preventing interference from variable redundancy and promoting more effective learning of individual characteristics. The process is illustrated in Figure S3 PatchTST initially models each variable channel independently to extract its temporal features, subsequently generating the overall prediction output by concatenating the predictions from all channels.

#### Patching strategy

PatchTST employs an innovative segmentation strategy that divides raw time series data into multiple fixed-length patches, effectively reducing sequence length and enhancing modeling efficiency. Let the original sequence be denoted as $x\left(t\right)\in R^{L}$, where *L* represents the number of time steps. The time series is first segmented into multiple non-overlapping patches of fixed length *P*. Figure S4 illustrates the specific segmentation process, which proceeds as follows:

(Equation 39) $$x\left(t\right)=\left\{x^{\left(1\right)},x^{\left(2\right)},\ldots ,x^{\left(N\right)}\right\},x^{\left(i\right)}\in R^{P},i=1,2,\ldots ,N$$

Here, $N=\lfloor \frac{L}{P}\rfloor$ denotes the total number of patches, each of length *P*, covering the time window of the original sequence.

#### Patch embedding and temporal order

The patch-embedding module maps each time-series patch into a high-dimensional representation, producing the token sequence consumed by the Transformer. For each partitioned patch $x^{\left(i\right)}\in R^{F}$, it is mapped to a high-dimensional space via linear projection:

(Equation 40) $${\hat{x}}^{\left(i\right)}=W_{p}\cdot x^{\left(i\right)}+b_{p}$$

Here, $W_{p}\in R^{d\times P}$ represents the linear mapping matrix, $b_{p}\in R^{d}$ is the bias term, and ${\hat{x}}^{\left(i\right)}\in R^{d}$ is the patch representation after embedding, with *d* being the dimension after mapping. As shown in Figure S5, PatchTST simultaneously captures local patterns and global dependencies by partitioning each time series into multiple patches and performing embedding through a self-attention mechanism.

#### Transformer encoder for patch token

The encoder architecture of the PatchTST model is based on the standard Transformer framework.^20^ After segmenting the input time series into patches, each patch is treated as a token and projected into an embedding space. These patch tokens are then fed into a stack of Transformer encoder layers consisting of multi-head self-attention and position-wise feedforward networks with residual connections and layer normalization. This architecture enables information exchange among patches and allows the model to capture both local patterns within patches and long-range temporal dependencies across the sequence. As the Transformer encoder formulation is standard and widely used, we only provide this brief description here and refer the reader to^20^ for more details.

#### Prediction head

The PatchTST model introduces a concise and efficient prediction head that operates atop the encoder output, transforming high-dimensional patch representations into target predictions. The design of this prediction module fully considers the continuity and numerical regression characteristics inherent in time series forecasting. It employs a fully connected layer as the mapping function to achieve the transformation from the representation space to the target space. The Transformer encoder outputs high-dimensional feature vectors for each patch token, denoted as follows:

(Equation 41) $$Z=\left[z_{1},z_{2},\ldots ,z_{N}\right]\in R^{N\times d}$$

Here, *N* denotes the number of patches, and *d* represents the encoder dimension. The prediction head applies a linear transformation to each *z*_*i*_, mapping it to generate the corresponding prediction output.

(Equation 42) $${\hat{y}}_{i}=z_{i}\cdot W+b,i=1,2,\ldots ,N$$

Here, $W\in R^{d\times 1}$ is defined as the learnable weight matrix, while b∈R denotes the associated bias parameter. To maintain consistency in the sequence structure, PatchTST concatenates all patch outputs $\hat{y}i$ in chronological order to form the final result. $\hat{Y}$ represents the regression output for consecutive time steps:

(Equation 43) $$\hat{Y}=\left[{\hat{y}}_{1},{\hat{y}}_{2},\ldots ,{\hat{y}}_{N}\right]$$

#### ProbSparse-PatchTST

Classic self-attention demonstrates *O(L*^*2*^*)* time and space complexity for sequences of length *L*. In industrial time series, particularly those characterized by significant noise and non-stationarity (e.g., sulfur gas emission sequences), challenges such as the averaging of attention weights and the dilution of long-range dependencies by noise frequently occur. PatchTST effectively reduces token count through a patching mechanism while maintaining the local temporal structure. However, the encoder still necessitates computationally intensive attention calculations across all queries. This work embeds a sparse probabilistic self-attention module into the PatchTST encoder, computing attention only for queries identified as probabilistically salient. This innovative approach not only compresses computational resources but also effectively suppresses the propagation of noise.

Linearly map the *i-th* patch sequence $x_{P}^{\left(i\right)}\in R^{P\times N}$ to the Transformer latent space, and incorporate learnable positional encodings to preserve temporal order:

(Equation 44) $$x_{d}^{\left(i\right)}=W_{P}x_{P}^{\left(i\right)}+W_{\text{POS}},x_{d}^{\left(i\right)}\in R^{D\times N}$$

Where $W_{P}\in R^{D\times P}$ denotes the trainable projection matrix, $W_{\text{POS}}\in R^{D\times N}$ represents the learnable positional encoding, *P* is the patch length, *N* denotes the number of input channels, while *D* represents the dimensionality of the latent space.

In the *h-th* attention head, the query matrix $Q_{h}^{\left(i\right)},K_{h}^{\left(i\right)},V_{h}^{\left(i\right)}\in R^{D_{h}\times N}$ provides the standard output:

(Equation 45) $${\left(O_{h}^{\left(i\right)}\right)}^{⊤}=\text{Softmax}\left(\frac{{\left(Q_{h}^{\left(i\right)}\right)}^{⊤}K_{h}^{\left(i\right)}}{\sqrt{d_{k}}}\right)V_{h}^{\left(i\right)},d_{k}=D_{h}$$

The probabilistic score based on KL divergence approximates the dominance of query vectors over attention distributions. For the *i-th* query *q*_*i*_, it is defined as:

(Equation 46) $$M\left(q_{i},K\right)=\ln\left(\sum_{j=1}^{L_{K}}e^{\frac{q_{i}k_{j}^{⊤}}{\sqrt{d}}}\right)-\frac{1}{L_{K}}\sum_{j=1}^{L_{K}}\frac{q_{i}k_{j}^{⊤}}{\sqrt{d}},d=d_{k}$$

In this context, *k*_*ij*_ refers to the *j*-th of K (the key matrix), while *L*_*k*_ indicates the number of keys. The first term corresponds to log∑exp (emphasizing peak responses), while the second term represents mean correction (penalizing suboptimal alignments). Their difference quantifies the query’s tendency to generate a sharp attention distribution. The top-*u* queries for each head are retained to form the sparse set $\bar{Q}$, with the remaining query channels set to zero (preserving only residual paths) to reduce redundant computations and mitigate noise propagation.

Perform attention aggregation on the filtered $\bar{Q}$ (scaling and normalization consistent with the baseline):

(Equation 47) $${\left(O_{h}^{\left(i\right)}\right)}^{⊤}=\text{Softmax}\left(\frac{{\left({\bar{Q}}_{h}^{\left(i\right)}\right)}^{⊤}K_{h}^{\left(i\right)}}{\sqrt{d_{k}}}\right)V_{h}^{\left(i\right)}$$

Subsequently, the feature $Z^{\left(i\right)}\in R^{D\times N}$ is obtained through the parallel connection of multiple heads, residual connections, layer normalization, and a feedforward network, with the final predictions produced by the linear readout layer.

#### Flowchart of the proposed VMD-CEEMDAN-PIMO-PatchTST model

Building upon the aforementioned methods, we propose a sulfur gas concentration prediction model that integrates VDES quadratic decomposition, sample entropy reconstruction, the PIMO optimization algorithm, and the PatchTST-PSA model. Figure 1 presents the overall framework of the model, with the subsequent steps described as follows.

Step 1: This study collects the measured concentration sequences of sulfur dioxide (SO_2_) and hydrogen sulfide (H_2_S) during the Sulfur Recovery Unit (SRU) process. The raw sequences are adaptively decomposed using VMD to obtain multi-scale Intrinsic Mode Functions (IMFs) and residuals (*r*).
Step 2: Input the residuals *r* from VMD into CEEMDAN for secondary decomposition to obtain a finer-grained IMF family. Then, calculate the sample entropy for the original sequence, each VMD-IMF, and each CEEMDAN-IMF. Perform complexity-constrained reconstruction by aggregating subcomponents with sample entropy values lower than that of the original sequence into reconstructed IMF groups based on a threshold, thereby reducing dimensionality and noise. Retain the remaining high-complexity components separately to emphasize key dynamic features.
Step 3: The input tensor consists of the reconstructed Intrinsic Mode Function (IMF) components along with the available environmental variables. The architecture of PatchTST-PSA, as well as the hyperparameter search space, are clearly defined, alongside the training partition, objective function, and early stopping criterion.
Step 4: Employing PIMO for projection-iterative joint optimization within a defined search space effectively balances global exploration with local refinement, resulting in optimal hyperparameter combinations.
Step 5: Train PatchTST-PSA using the optimal hyperparameters identified in Step 4 to generate prediction sequences for each reconstruction component. The ProbSparse attention mechanism is applied exclusively to the dominant query, thereby preserving essential long-range dependencies while simultaneously reducing computational complexity.
Step 6: The final predictions for SO_2_ and H_2_S were derived by linearly combining the component predictions in accordance with the established reconstruction rules. Subsequently, evaluation metrics, including RMSE, MAE, MAPE, and R^2^, were computed to assess the accuracy of these predictions.

#### Data description

The dataset utilized in this study is derived from the task of predicting SO_2_ and H_2_S concentrations for the fourth line tail gas of the Italian SRU plant, as detailed by Fortuna et al.^40^ The SRU process effectively converts hydrogen sulfide (H_2_S) present in acid gases into elemental sulfur. This conversion not only mitigates environmental pollution but also facilitates the recovery of sulfur as a valuable resource. The process involves several variables, including gas flow (MEA_GAS), air flow (AIR_MEA), secondary air flow (AIR_MEA_2), SWS zone gas flow (SWS_GAS_TOT), and SWS zone air flow. Understanding the interplay of these variables is crucial for optimizing the SRU process and enhancing its efficiency. MEA gas and SWS gas undergo partial oxidation reactions with air in the reactor, resulting in the formation of intermediate sulfur products. These products are subsequently converted through a catalytic converter. The resulting sulfur liquid is collected and cooled, while the residual H_2_S and SO_2_ in the exhaust gas undergo additional treatment. The process is illustrated in Figure 2. Samples were collected at 1-min intervals, with each sample comprising five input variables and two output variables, namely the concentrations of SO_2_ and H_2_S. In this experiment, the initial 10,081 samples from the dataset were selected, with 70% allocated for the training set and the remaining 30% designated as the test set. Detailed information regarding the dataset is presented in Table 1.

Figure S6 illustrates the SO_2_ and H_2_S concentration time series, showing significant high-frequency noise. These high-frequency fluctuations may arise from various sources, including measurement noise, sensor inaccuracies, and fast process disturbances within the SRU plant. The presence of these rapid fluctuations introduces challenges in the accurate prediction of sulfur gas concentrations. As shown in Figure S6, the noisy data can be seen as sudden, sharp spikes in the time series, making it difficult to distinguish between actual signal variations and noise.

#### Data processing

##### Primary decomposition

Variational Mode Decomposition (VMD) is an adaptive signal processing method based on a variational framework that decomposes non-stationary signals into multiple bandwidth-limited and mutually independent intrinsic modal functions (IMFs). By minimizing the combined bandwidths of each mode, VMD effectively separates different frequency components, demonstrating remarkable noise reduction performance and robustness. In comparison to Empirical Mode Decomposition (EMD) and its variants, VMD permits predefining the number of modes *K*. This feature enhances both controllability and stability while effectively managing computational complexity. The selection of *K* significantly influences the quality of decomposition. An excessively small *K* may lead to mode aliasing and information loss, thereby diminishing modeling accuracy. Conversely, an excessively large *K* may result in over-decomposition, introducing redundant components. This work determines the optimal decomposition depth for the SRU sulfur gas concentration time series by adopting the signal-to-noise ratio (SNR) as the metric. Decompositions are performed sequentially for *K* values ranging from 2 to 10, calculating the SNR of the reconstructed signal at each *K*. The maximum SNR value obtained is then selected as the optimal mode number.

Figure 3(left) illustrates the signal-to-noise ratio across various values of *K*. Within the range of *K* ∈ [2, 10], the optimal number of decomposed components for the H_2_S and SO_2_ concentration sequences is attained when *K* = 10. The right panel of Figure 3 illustrates the intrinsic mode function (IMF) components and the residual components obtained from the VMD at *K* = 10. The residual sequence produced by VMD is notably large and contains significant white noise. This work addresses the residual component by applying CEEMDAN for a second-stage decomposition.

##### Secondary decomposition

The rigorous determination of the CEEMDAN parameters, namely the ensemble size *N* and the noise amplitude *ϵ*, is essential to ensure the robustness and reliability of the decomposition. The optimal value of *N* was determined by fixing *ϵ* = 0.05 and analyzing the stability of the decomposition structure, characterized by the number of intrinsic mode functions (IMFs, *K*), as well as the decomposition characteristics, represented by the average center frequency (*f*), over the range *N* ∈ [20,200]. As illustrated in Figure 4A, although minor fluctuations were observed at *N* = 60 and *N* = 120, the modal count remained stable at *K* = 11 IMFs, and the average center frequency varied within a narrow and highly convergent range from 0.0358 to 0.0365; accordingly, *N* = 100 was selected, as it lies within the stable region for *K* and ensures feature convergence with an acceptable computational burden. Subsequently, the noise amplitude ϵwas determined by fixing *N* = 100 and evaluating the mean Orthogonality Index (OI) and its standard deviation (*σ*) as indicators of decomposition quality and stability, respectively, with the analysis repeated *M* = 10 times. As shown in Figure 4B, the average OI attains its minimum value of 0.5507 at *ϵ* = 0.01, indicating the best decomposition quality and highest stability; therefore, *ϵ* = 0.01 was adopted for the subsequent experiments.

This paper introduces CEEMDAN for the secondary decomposition of residual components obtained from VMD. In the CEEMDAN procedure, the ensemble size is set to 100, the noise amplitude is fixed at 0.01, and the number of decomposition modes is determined adaptively. Taking the H_2_S concentration sequence as an example, the VMD residual is further decomposed into 11 intrinsic mode functions (IMFs). Using the same procedure, the CEEMDAN decomposition results for the residual sequence of SO_2_ concentration can be obtained accordingly.

The secondary VMD-CEEMDAN decomposition produces a large number of IMF components across different temporal scales. Directly modeling all decomposed components would significantly increase computational complexity and may introduce error accumulation during signal reconstruction. To address this issue, sample entropy (SE) is introduced as a complexity-based criterion to guide component selection and aggregation, thereby reducing the number of modeling sub-sequences while preserving the essential dynamical characteristics of the original signal.

As shown in Figure 5, the entropy distributions of VMD and CEEMDAN IMFs exhibit clear stratification, with the selected thresholds located in low-density transition regions between structured and noise-dominated components. This indicates that the thresholds are data-adaptive and not arbitrarily chosen. The robustness of the entropy-guided reconstruction with respect to threshold variations is further examined through sensitivity analysis in Section 4.1.2.

Based on the quantitative entropy statistics summarized in Table 2, a two-tier entropy-guided reconstruction strategy is adopted. For VMD-derived IMFs, components with SE values lower than the sample entropy of the original sequence are regarded as relatively regular signal components and aggregated into a unified modeling sub-sequence, while the remaining components are retained for individual modeling. For CEEMDAN-derived IMFs obtained from the VMD residual, components with SE values lower than the residual entropy threshold are aggregated as weak but structured features, whereas higher-entropy components are identified as noise-dominated and discarded. The reconstructed IMF components are illustrated in Figure 5.

#### Model hyperparameters

To enhance the prediction performance of sulfur-gas concentrations, grid search and random search were first employed to identify reasonable ranges for the key hyperparameters. Based on these ranges, the PIMO algorithm was subsequently applied to optimize the proposed PatchTST-PSA model. The optimal hyperparameters obtained through the PIMO optimization process, together with the basic parameter settings of all comparative models, are summarized in Table S1. Using these configurations, H_2_S and SO_2_ concentrations were predicted with the proposed model and several baseline methods, including BP, GRU, LSTM, Transformer, Informer, PatchTST, VMD-PatchTST, and VDES-PatchTST.

### Quantification and statistical analysis

To comprehensively assess predictive accuracy, this study employs four standard metrics: root-mean-square error (RMSE), mean absolute error (MAE), mean absolute percentage error (MAPE), and the coefficient of determination (R^2^). The corresponding formulas are given in Equations 48, 49, 50, and 51. Here, *n* denotes the total number of data points in the forecast, while *y*_*i*_, ${\hat{y}}_{i}$, and $\bar{y}$ represent the actual value, the forecasted value, and the mean of the actual values, respectively.

(Equation 48) $$\text{RMSE}=\sqrt{\frac{1}{n}\sum_{i=1}^{n}{\left(y_{i}-\hat{y_{i}}\right)}^{2}}$$

(Equation 49) $$\text{MAE}=\frac{1}{n}\sum_{i=1}^{n}\left|y_{i}-{\hat{y}}_{i}\right|$$

(Equation 50) $$\text{MAPE}=\frac{100\%}{n}\sum_{i=1}^{n}\left|\frac{y_{i}-{\hat{y}}_{i}}{y_{i}}\right|$$

(Equation 51) $$R^{2}=1-\frac{\sum_{i=1}^{n}{\left(y_{i}-{\hat{y}}_{i}\right)}^{2}}{\sum_{i=1}^{n}{\left(y_{i}-\bar{y}\right)}^{2}}$$

MAE is a fundamental metric for assessing prediction errors, measuring the average absolute deviation between predicted values and actual observed values. This metric provides an intuitive reflection of the overall level of prediction error. Building upon this, the MAPE introduces the concept of relative error by considering the proportion of error in relation to the actual value, thereby enhancing the comprehensive evaluation capability of model prediction performance. The RMSE is particularly sensitive to larger errors, effectively capturing the volatility and dispersion of prediction results. In general, lower values of MAE, MAPE, and RMSE signify a higher level of prediction accuracy for the model. Additionally, R^2^, as a measure of model goodness-of-fit, ranges from 0 to 1, with values closer to 1 indicating a stronger ability of the model to explain the actual data.
