# Supplementary material for: Hybrid multiscale forecasting of SRU sulfur gas concentrations using VMD CEEMDAN and optimized PatchTST
Source: iScience. 2026 Feb 11;29(3):114986. doi: 10.1016/j.isci.2026.114986 (PMC12993888; doi:10.1016/j.isci.2026.114986)
Supplement: Document S1. Figures S1–S10 and Tables S1–S5 [file mmc1.pdf]

iScience, Volume *29*

Supplemental information

Hybrid multiscale forecasting of SRU sulfur gas concentrations using VMD CEEMDAN and optimized PatchTST

Wenzhe Sun, Longhao Li, Binglin Lu, Lijun Jiang, and Jie Zhang

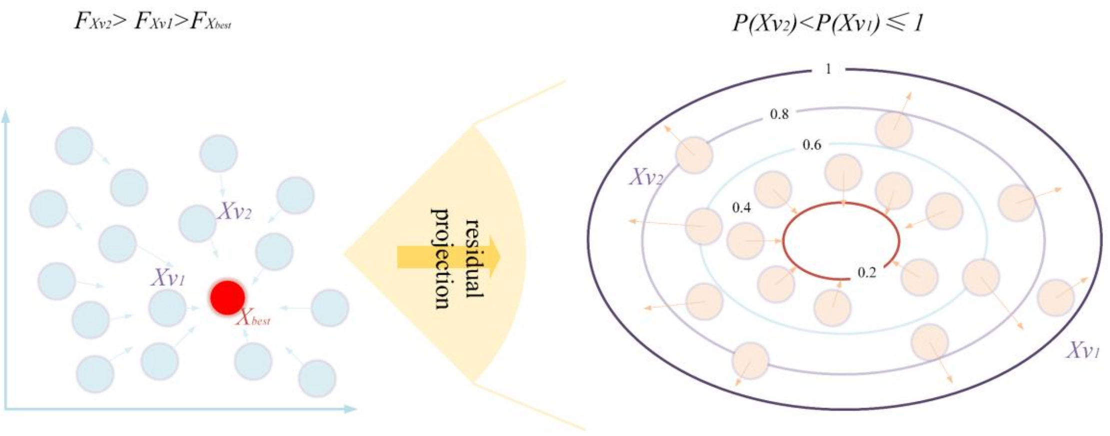

Figure S1. Schematic of Residual-Guided Projection Workflow Candidate solutions are updated by projecting residual directions around X_{best} based on fitness ranking $F(X_{v2}) > F(X_{v1}) > F(X_{best})$. The adaptive search radius is controlled by the acceptance probability $P(\cdot)$

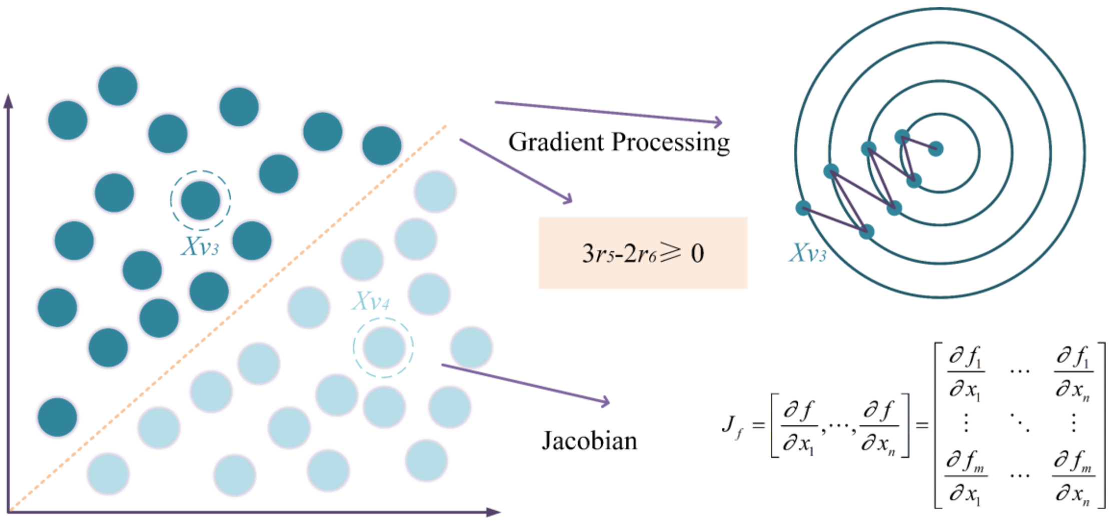

$$J_f = \left[\frac{\partial f}{\partial x_1}, \cdots, \frac{\partial f}{\partial x_n}\right] = \begin{bmatrix} \frac{\partial f_1}{\partial x_1} & \cdots & \frac{\partial f_1}{\partial x_n} \\ \vdots & \ddots & \vdots \\ \frac{\partial f_m}{\partial x_1} & \cdots & \frac{\partial f_m}{\partial x_n} \end{bmatrix}$$

Figure S2. Dual Random Projection Workflow with Gradient-Correction Branch Candidates X_{v3}, X_{v4} are generated by random projection, and the Jacobian J_f is used for gradient correction to refine the update direction.

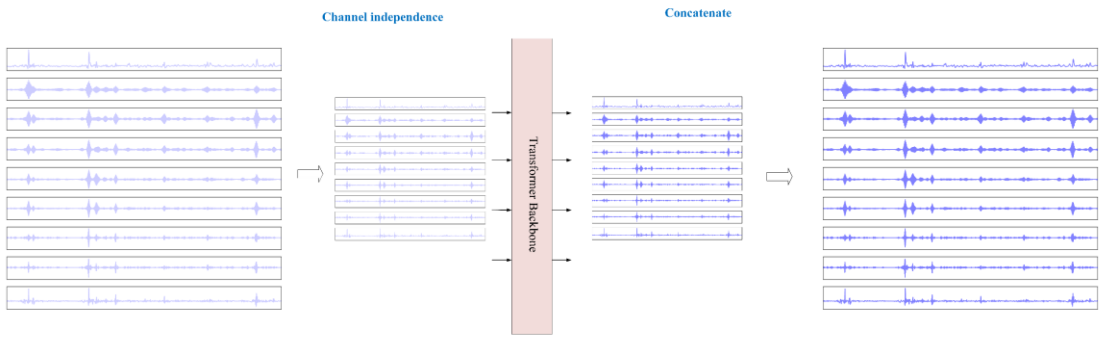

Figure S3. Channel independence diagram of PatchTST Univariate series are processed independently with a shared Transformer backbone and then concatenated, mitigating inter-channel interference.

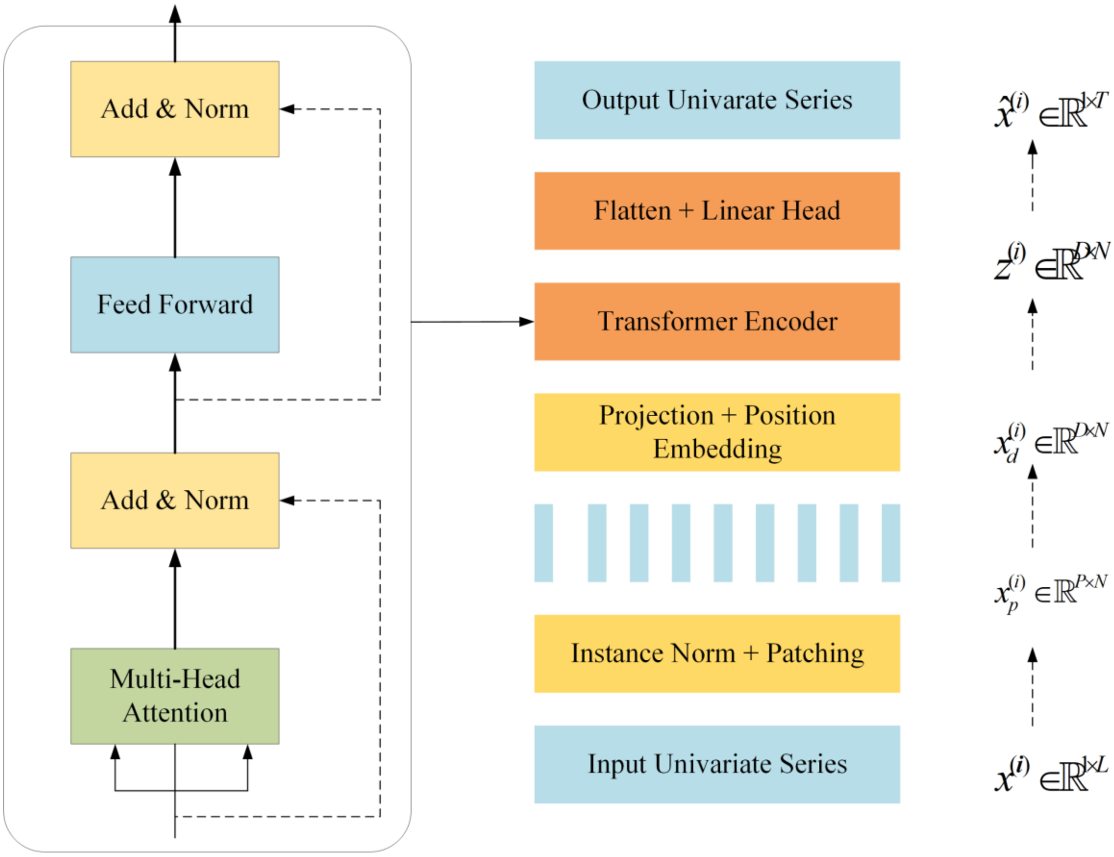

Figure S4. The patch operation of PatchTST An input univariate series $x^{(i)}$ is normalized and segmented into patches $x^{(i)}{}_p$, then mapped by projection and positional embedding before being processed by the Transformer encoder. The encoded representation is flattened and passed to a linear head to produce the forecast $\hat{x}^{(i)}$.

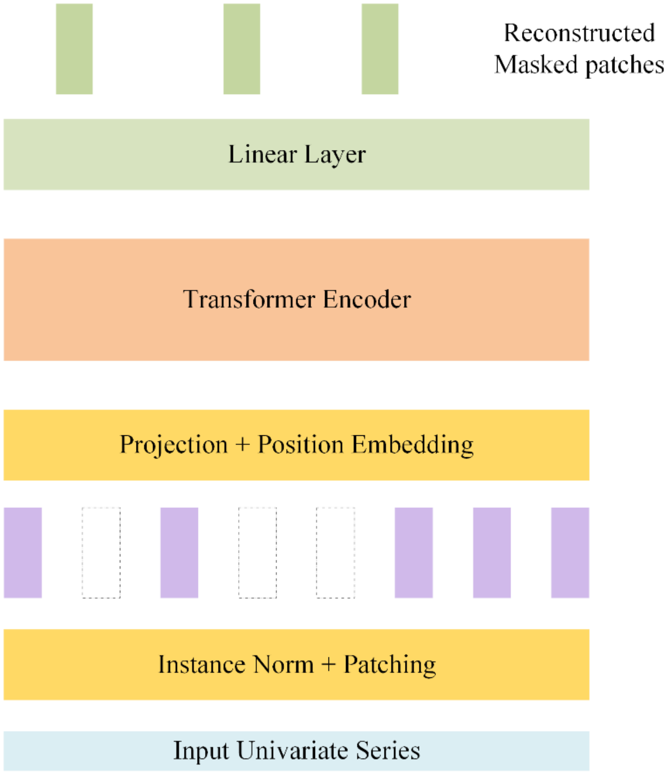

Figure S5. Self-supervised learning A subset of input patches is masked, and the model is trained to reconstruct them, yielding transferable temporal representations for later fine-tuning.

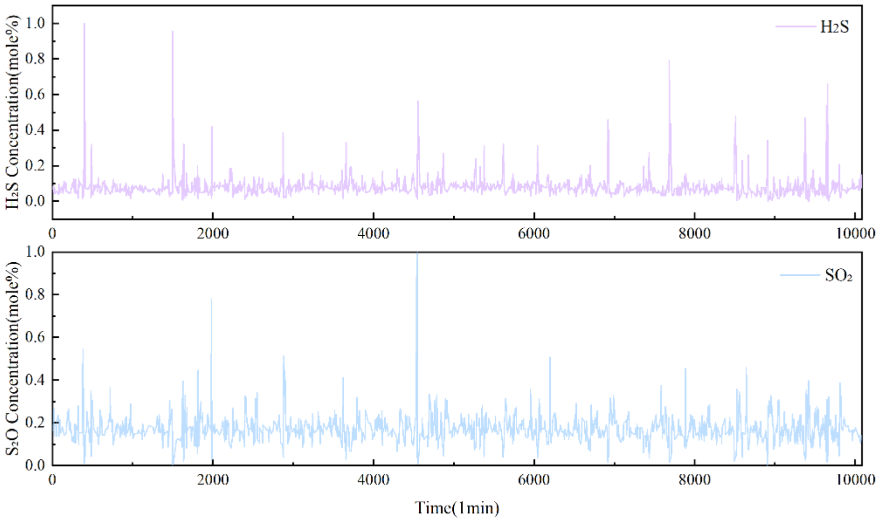

Figure S6. Raw H_2S and SO_2 concentration signals The figure shows the unprocessed H_2S and SO_2 concentration signals (mol%) at 1-min resolution over the full recording period used in this study.

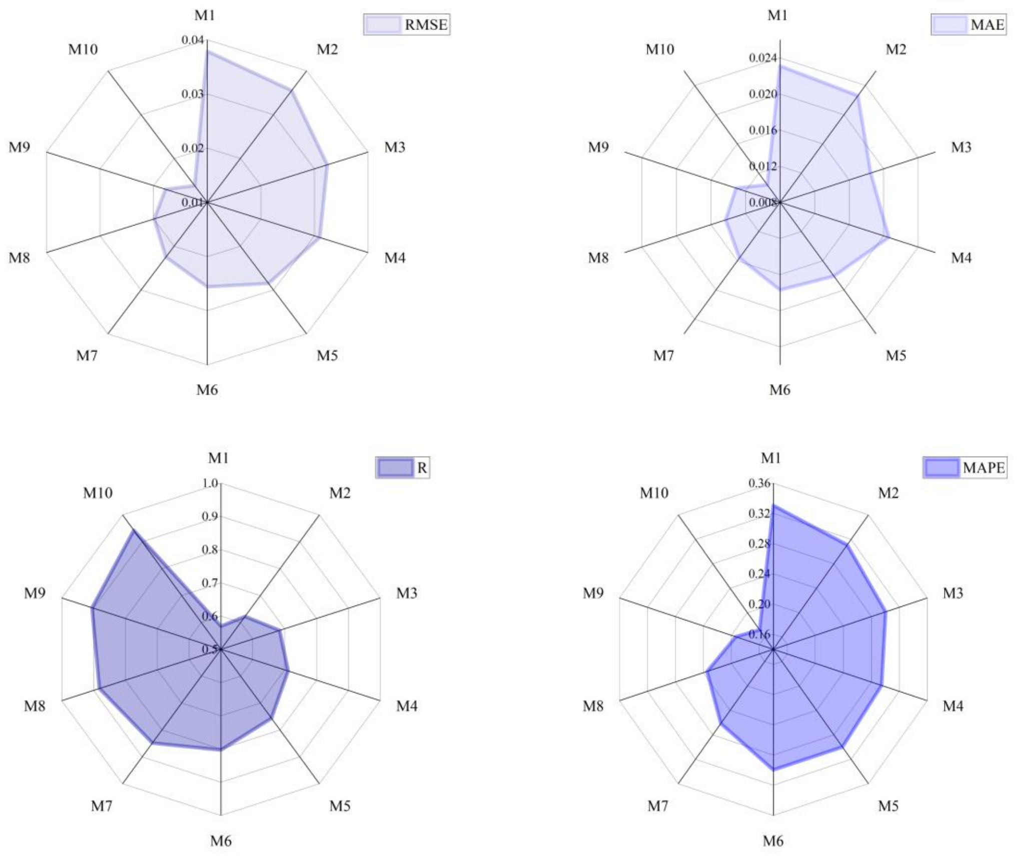

Figure S7. Comparison of RMSE, MAE, MAPE, and R metrics for different models on the H_2S dataset Radar charts summarize RMSE, MAE, MAPE, and R for models M1–M10. Lower values indicate better performance for RMSE/MAE/MAPE, whereas higher values indicate better performance for R.

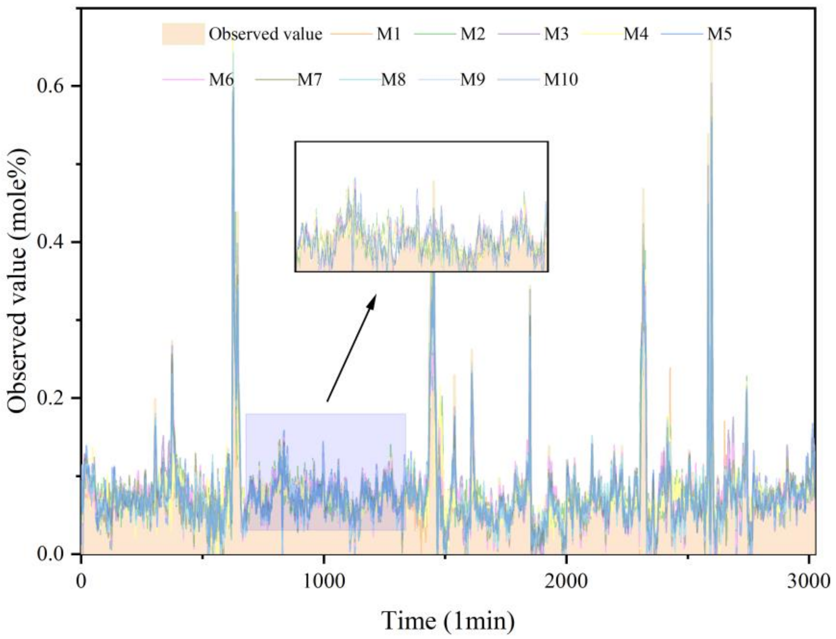

Figure S8. Comparison of predicted and observed H_2S concentration curves over time Observed concentrations are compared with predictions from models M1 – M10 over the same time window. The inset zooms into the highlighted segment, showing that M10 follows the high-frequency variations and peak events more closely.

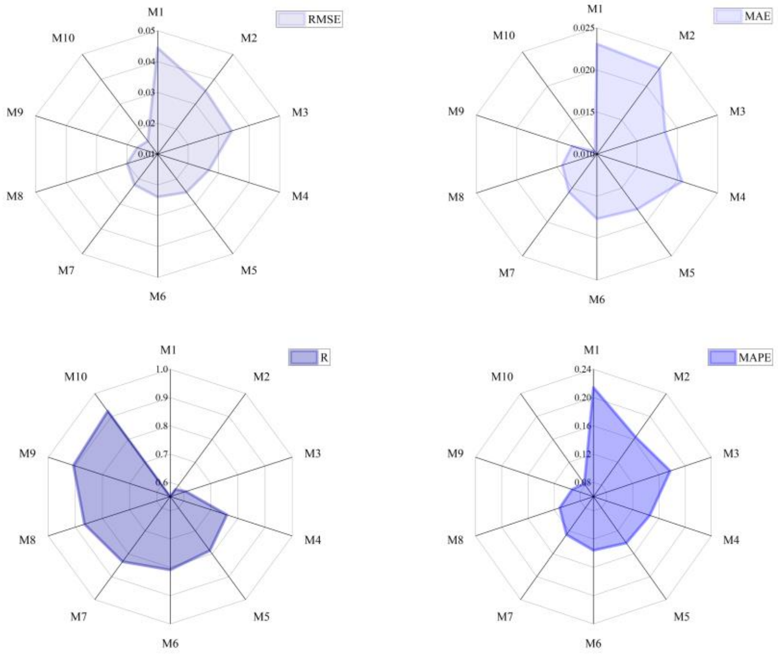

Figure S9. Comparison of RMSE, MAE, MAPE, and R metrics for different models on the SO_2 dataset. Radar charts summarize RMSE, MAE, MAPE, and R for models M1–M10. Lower values indicate better performance for RMSE/MAE/MAPE, whereas higher values indicate better performance for R.

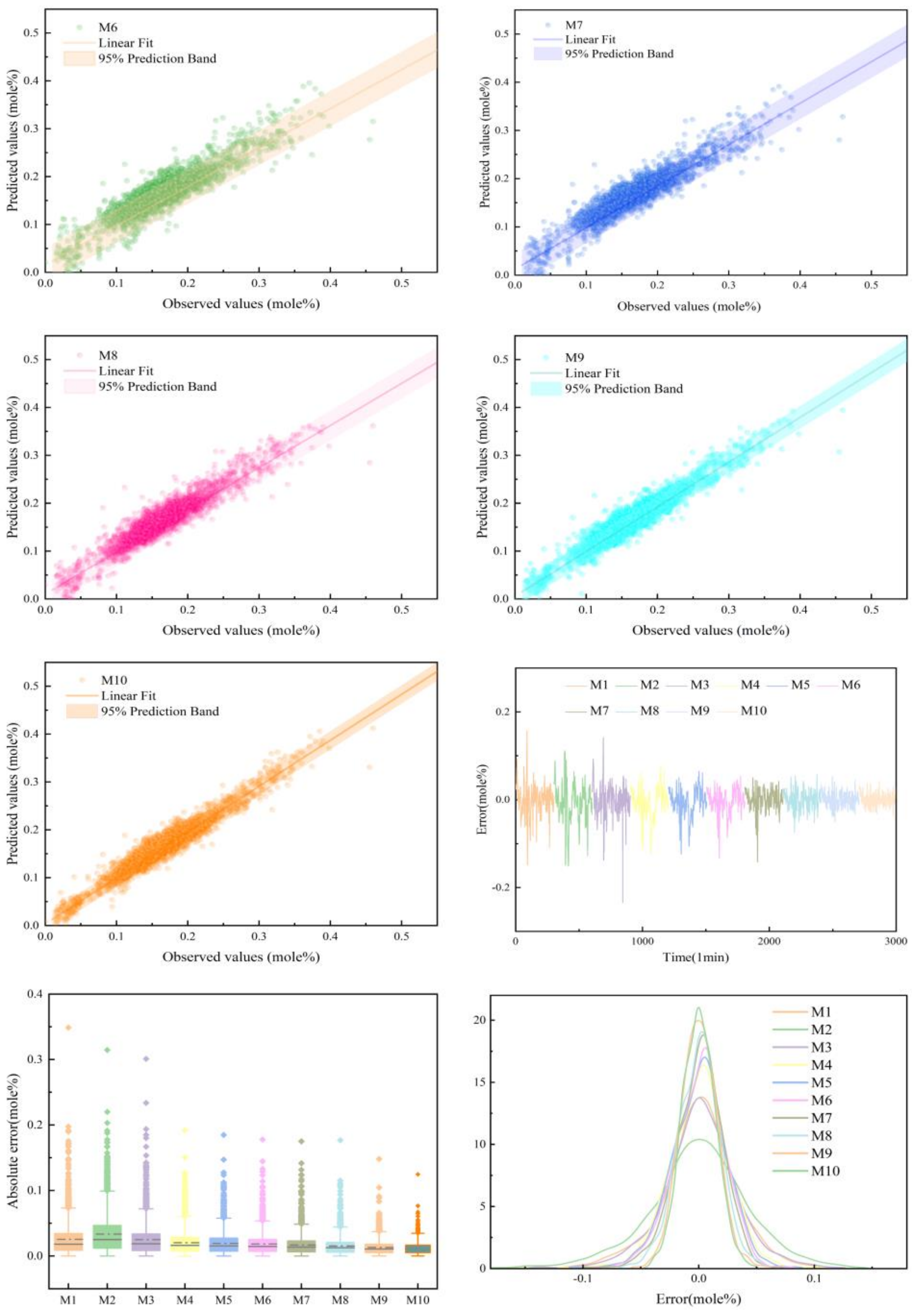

Figure S10. Regression fitting and error analysis of different models on the SO_2 dataset Scatter plots show predicted versus observed SO_2 concentrations for models M6–M10 with linear fits; shaded regions indicate the 95% prediction bands of the fitted regression (top panels). The error time series for all models is shown in the middle-right panel. Boxplots summarize the absolute-error distribution across the test samples (center line, median; box, interquartile range; whiskers, 1.5× IQR; points, outliers). The bottom-right panel shows the kernel density estimates of prediction errors for models M1 – M10.

Table S1. Basic parameter settings for all models Summary of key hyperparameter settings for models M1 - M10 used in this study.

Model	Abbr.	Parameter value settings
Proposed model	M10	seq_len=960, pred_len=60, patch_len=24, stride=6, d_model=128, n_head=8, n_layer=3, top_u=0.5, lr=1e-4.
BP	M1	hidden_layer=[2,3],hidden_units=[64,256],activation=ReLU,optimizer=Adam,learning_rate=5e-4,weight_decay=1e-4.
GRU	M2	Input_window=720,forecast_horizon=60,hidden_size=[64,256],num_layers=[1,3],dropout=0.1,learning_rate=1e-4.
LSTM	M3	seq_len=720,pred_len=60,num_layer=[2,4],weight_decay=1e-4,optimizer=Adam,hidden_size=[128,384].
Transformer	M4	seq_len=960,pred_len=60,d_model=128,n_head=8,n_layer=3,d_ff=512,dropout=0.1
Informer	M5	seq_len=960,pred_len=60,label_len=480,d_model=128,n_head=8, e_layers=3.
PatchTST	M6	seq_len=720,pred_len=60,patch_len=20,stride=5,d_model=128,n_heads=8,m_layers=3,learning_rate=1e-4,weigh_decay=1e-4.
PatchTST-PSA	M7	Parameter settings are the same as the proposed model.
VMD-PatchTST-PSA	M8	Parameter settings are the same as the proposed model.
VDES-PatchTST-PSA	M9	Parameter settings are the same as the proposed model.

Table S2. Robustness analysis of entropy thresholds Evaluation of sensitivity to the entropy threshold γ in the subsequence decomposition step. The table reports Seraw, Seres, the number of reconstructed subsequences, and the resulting forecasting metrics (RMSE and R^2)

γ	SE_raw*	SE_res*	nSubseq_total	RMSE	R^2
0.8	0.1683	0.3466	8	0.0138	0.9429
0.9	0.1894	0.3899	8	0.0138	0.9429
1.0	0.2104	0.4332	8	0.0138	0.9429
1.1	0.2314	0.4765	7	0.0146	0.9380
1.2	0.2525	0.5198	6	0.0155	0.9320

Table S3. A brief computational cost comparison of different model variants Comparison of computational cost among single-stage and multi-stage model variants. The table reports the number of subsequences, training time (s/epoch), peak GPU memory during training (MB), and inference time (ms/sample).

Model	Model stage	Subsequences	Train time (s/epoch)	Peak GPU Memory(train,MB)	Infer time (ms/sample)
PatchTST	Single-stage	1	3.0308±0.0763	770.5	0.033999
PatchTST-PSA	Single-stage	1	4.1931±0.6454	560.0	0.059526
VDES-PatchTST-PSA	Multi-stage	8	2.9474±0.4203	291.61	0.060892
VMD-CEEMDAN-PIMO-PatchTST-PSA (without entropy aggregation)	Multi-stage	21	2.8498±0.2206	297.53	0.051131
VDES-PIMO-PatchTST-PSA	Multi-stage	8	5.2799±0.1746	348.45	0.060414

Table S4. Comparison of SO2 concentration predictions Quantitative evaluation of models M1 – M10 on the SO_2 dataset using RMSE, MAE, R^2, and MAPE. Lower RMSE/MAE/MAPE and higher R^2 indicate better performance; the best value in each column is shown in bold.

Model	Model ID	RMSE (mole%)	MAE (mole%)	R^2	MAPE (%)
BP	M1	0.0444	0.0331	0.4420	0.2143
GRU	M2	0.0354	0.0253	0.5813	0.1640
LSTM	M3	0.0343	0.0256	0.6079	0.1772
Transformer	M4	0.0268	0.0202	0.7593	0.1460
Informer	M5	0.0253	0.0192	0.7861	0.1411
PatchTST	M6	0.0239	0.0181	0.8091	0.1360
PatchTST-PSA	M7	0.0223	0.0167	0.8342	0.1265
VMD-PatchTST-PSA	M8	0.0200	0.0151	0.8657	0.1115
VDES-PatchTST-PSA	M9	0.0167	0.0129	0.9070	0.0925
VDES-PIMO-PatchTST-PSA	M10	**0.0152**	**0.0119**	**0.9228**	**0.0825**

Table S5. Comparison of Percentage Improvement for Various Models in Predicting H_2S and SO_2 Concentrations Percentage improvement of the proposed model (M10) over baseline models (M1 – M9) on the H_2S and SO_2 datasets. P_{RMSE}, P_{MAE}, and P_{MAPE} denote the relative reduction (%) of each metric compared with the corresponding baseline (higher values indicate larger improvement).

Case studies	Original models	P_{RMSE} (%)	P_{MAE} (%)	P_{MAPE} (%)
H_2S	BP	63.59%	54.98%	47.97%
	GRU	61.13%	53.98%	44.71%
	LSTM	57.41%	43.78%	42.81%
	Transformer	55.48%	49.51%	41.69%
	Informer	51.58%	42.54%	40.91%
	PatchTST	46.09%	41.24%	42.64%
	PatchTST-PSA	38.67%	33.33%	34.26%
	VMD-PatchTST-PSA	30.65%	27.27%	26.76%
	VDES-PatchTST-PSA	22.47%	20.61%	11.30%
SO_2	BP	65.77%	64.05%	61.50%
	GRU	57.06%	52.96%	49.70%
	LSTM	55.69%	53.52%	53.44%
	Transformer	43.28%	41.09%	43.49%
	Informer	39.92%	38.02%	41.53%
	PatchTST	36.40%	34.25%	39.34%
	PatchTST-PSA	31.84%	28.74%	34.78%
	VMD-PatchTST-PSA	24.00%	21.19%	26.01%
	VDES-PatchTST-PSA	8.98%	7.75%	10.81%
